# A simplified liquid chromatography‐mass spectrometry methodology to probe the shikimate and aromatic amino acid biosynthetic pathways in plants

**DOI:** 10.1111/tpj.17105

**Published:** 2024-10-28

**Authors:** Jorge El‐Azaz, Hiroshi A. Maeda

**Affiliations:** ^1^ Department of Botany University of Wisconsin‐Madison Madison Wisconsin USA

**Keywords:** shikimate pathway, aromatic amino acid biosynthesis, prephenate, arogenate, DAHP synthase, arogenate dehydrogenase, glyphosate, technical advance

## Abstract

Plants direct substantial amounts of carbon toward the biosynthesis of aromatic amino acids (AAAs), particularly phenylalanine to produce lignin and other phenylpropanoids. Yet, we have a limited understanding of how plants regulate AAA metabolism, partially because of a scarcity of robust analytical methods. Here, we established a simplified workflow for simultaneous quantification of AAAs and their pathway intermediates from plant tissues, based on extraction at two alternative pH and analysis by Zwitterionic hydrophilic interaction liquid chromatography coupled to mass spectrometry. This workflow was then used to analyze metabolic responses to elevated or reduced carbon flow through the shikimate pathway in plants. Increased flow upon expression of a feedback‐insensitive isoform of the first shikimate pathway enzyme elevated all AAAs and pathway intermediates, especially arogenate, the last common precursor within the post‐chorismate pathway of tyrosine and phenylalanine biosynthesis. Additional overexpression of an arogenate dehydrogenase enzyme increased tyrosine levels and depleted phenylalanine and arogenate pools; however, the upstream shikimate pathway intermediates remained accumulated at high levels. Glyphosate treatment, which restricts carbon flow through the shikimate pathway by inhibiting its penultimate step, led to a predictable accumulation of shikimate and other precursors upstream of its target enzyme but also caused an unexpected accumulation of downstream metabolites, including arogenate. These findings highlight that the shikimate pathway and the downstream post‐chorismate AAA pathways function as independently regulated modules in plants. The method developed here paves the way for a deeper understanding of the shikimate and AAA biosynthetic pathways in plants.

## Significance Statement

Aromatic amino acids (AAA) are key precursors to plant phenylpropanoids and other specialized metabolites. However, the regulation of AAA biosynthesis remains obscure partially due to the absence of robust methods for analyzing AAA pathway metabolites. Here, we report an extraction and LC–MS method for the simultaneous quantification of AAAs and their precursors from plant tissues. This methodology identified potentially regulated steps in the AAA pathways and will facilitate a deeper understanding of AAA metabolism.

## INTRODUCTION

**Figure 1:**
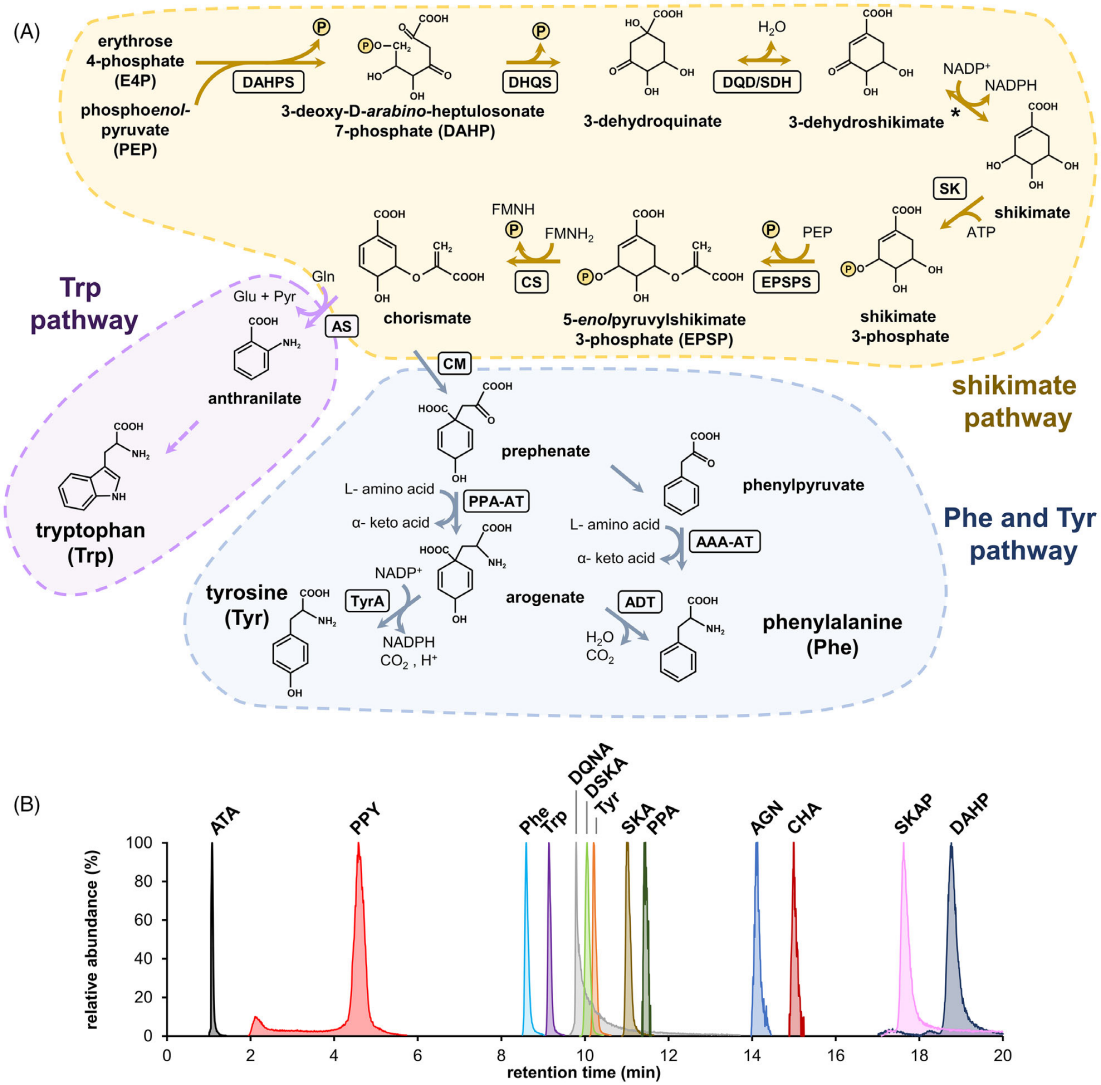
PEEK‐ZIC‐HILIC can resolve multiple intermediates of aromatic amino acid biosynthesis. (A) Overview of the pre‐ and post‐chorismate AAA biosynthetic pathways in plants. Note that the conversion of 3‐dehydroshikimate to shikimate (marked with an asterisk) is catalyzed by the bifunctional enzyme DQD/SDH. AAA‐AT, aromatic amino acid aminotransferase; ADT, arogenate dehydratase; AS, anthranilate synthase; CM, chorismate mutase; CS, chorismate synthase; DAHPS, 3‐deoxy‐D‐*arabino*‐heptulosoante 7‐phosphate (DAHP) synthase; DHQS, 3‐dehydroquinate synthase; DQD/SDH, bifunctional 3‐dehydroquinate dehydratase/shikimate dehydrogenase; EPSPS, 5‐*enol*pyruvylshikimate 3‐phosphate (EPSP) synthase; PPA‐AT, prephenate aminotransferase; SK, shikimate kinase; TyrA, arogenate dehydrogenase; Gln, glutamine; Glu, glutamate; Pyr, pyruvate. (B) Overlapped extracted ion chromatograms corresponding to authentic standards for the three AAAs and 11 of their biosynthetic precursors, resolved by PEEK‐ZIC‐HILIC and detected by electrospray ionization mass spectrometry (ESI/MS) in negative mode. ATA, anthranilate; AGN, arogenate; CHA, chorismate; DAHP, 3‐deoxy‐d‐*arabino*‐heptulosonate 7‐phosphate; DQNA, 3‐dehydroquinate; DSKA, 3‐dehydroshikimate; Phe, phenylalanine; PPA, prephenate; PPY, phenylpyruvate; SKA, shikimate; SKAP, shikimate 3‐phosphate; Trp, tryptophan; Tyr, tyrosine.

The plant aromatic amino acid (AAA) pathways link central carbon metabolism with the production of l‐phenylalanine (Phe), l‐tyrosine (Tyr), and l‐tryptophan (Trp) (Figure 1A), which are precursors to numerous plant natural products (Maeda & Dudareva, 2012; Tzin & Galili, 2010; Vogt, 2010). Among other compounds, the auxin plant hormone indole‐3‐acetic acid and indole glucosinolates are derived from Trp (Halkier & Gershenzon, 2006; Zhao, 2012), while tocopherols (vitamin E), plastoquinones and betalain pigments are synthesized from Tyr (Schenck & Maeda, 2018). Phe, typically the most abundant AAA in plant tissues, is precursor to phenylpropanoids, a structurally diverse and functionally multifaceted class of aromatic compounds that includes anthocyanins, flavonoids, and lignin (Boerjan et al., 2003; Bonawitz & Chapple, 2010; Vogt, 2010).

The production of AAAs in plants begins with a sequence of seven reactions known as the shikimate pathway, starting with the condensation of two central carbon metabolism intermediates, phospho*enol*pyruvate (PEP), and erythrose 4‐phosphate (E4P), into 3‐deoxy‐d‐*arabino*‐heptulosonate 7‐phosphate (DAHP) by the enzyme DAHP synthase (EC 2.5.1.54) (Figure 1A). Plant DAHP synthase enzymes are subjected to a complex feedback regulation mechanism by AAAs and other metabolites (El‐Azaz et al., 2023; Tzin et al., 2012; Yokoyama et al., 2021; Yokoyama, Kleven, et al., 2022; Yokoyama, de Oliveira, et al., 2022), which combined with transcriptional regulation (Lim et al., 2016; Weaver & Herrmann, 1997; Yokoyama, Kleven, et al., 2022), make DAHP synthase a key metabolic control point at the first step of AAA biosynthesis. Three additional enzymatic reactions convert DAHP into shikimate, after which this pathway is named. The next three enzymatic reactions convert shikimate to chorismate, the last common precursor to all three AAAs and precursor to salicylic acid (Lefevere et al., 2020; Maeda & Dudareva, 2012; Tzin & Galili, 2010). These latter steps include the enzyme *enol*pyruvylshikimate 3‐phosphate (EPSP) synthase (EC 2.5.1.19) (Figure 1A), a target of the widely used herbicide glyphosate (Steinrücken & Amrhein, 1980). Although some of the enzymatic activities of the shikimate pathway have been detected in the cytosol, the plastid is the sole compartment of the plant cell that, to date, has been shown to harbor all seven enzymatic steps to synthesize chorismate (Lynch, 2022; Lynch & Dudareva, 2020; Maeda & Dudareva, 2012; Weaver & Herrmann, 1997).

From chorismate, product of the linear shikimate pathway, two independent post‐chorismate pathways branch toward the synthesis of Trp on one side, and Tyr and Phe on the other. Trp is synthesized by six enzymatic steps, beginning with the conversion of chorismate into anthranilate (Figure 1A). In the case of Phe and Tyr, most plant species, including Arabidopsis, produce these two AAAs within the plastids via conversion of chorismate into prephenate and a subsequent transamination reaction that renders arogenate, the immediate precursor of both Tyr and Phe (Figure 1A) (Cho et al., 2007; Dal Cin et al., 2011; Graindorge et al., 2010; Maeda et al., 2010, 2011; Rippert et al., 2009). Although the majority of Phe is produced via arogenate within the plastids (Corea, Bedgar, et al., 2012; Corea, Ki, et al., 2012; Maeda et al., 2010, 2011; Rippert et al., 2009), plants also have a cytosolic pathway that produces Phe from phenylpyruvate (Figure 1A) (Eberhard et al., 1996; Lynch & Dudareva, 2020; Qian et al., 2019; Yoo et al., 2013). Additionally, the species in the legume family have an alternative pathway for producing Tyr from 4‐hydroxyphenylpyruvate in the cytosol (Gamborg & Keeley, 1966; Rubin & Jensen, 1979; Schenck et al., 2015, 2017). Thus, although the core steps of the shikimate and the post‐chorismate pathways have been well established, these alternative routes signify the diversity and compartmentalization of plant AAA biosynthesis.

Despite the importance of plant AAA pathways in supplying the precursors to multiple, and in some cases also abundant, plant‐specialized metabolites that respond dynamically to varied stimuli (Dong & Lin, 2021; Vogt, 2010), there is a prevalent shortage of analytical techniques for the study of AAA biosynthesis itself. Available methods are often indirect, time‐consuming, highly targeted to a specific compound, and not well suited for analyzing other metabolites. To overcome this critical roadblock, the present study aimed to develop a simpler liquid chromatography‐mass spectrometry (LC–MS) analytical approach to quantify AAA biosynthetic intermediates, particularly from plant tissues. To this end, we took advantage of Zwitterionic hydrophilic interaction liquid chromatography (ZIC‐HILIC), a highly versatile LC–MS column chemistry for targeting polar compounds (Buszewski & Noga, 2012; McCalley, 2017). This resulted in an improved extraction method and LC–MS analysis that enabled the simultaneous quantification of AAAs and their biosynthetic precursors from plant tissues, also being compatible with the detection of many other plant metabolites. Analysis of *Arabidopsis thaliana* and *Nicotiana benthamiana* plants expressing feedback‐deregulated isoforms of the first enzyme of the shikimate pathway, DAHP synthase (El‐Azaz et al., 2023; Yokoyama, Kleven, et al., 2022; Yokoyama, de Oliveira, et al., 2022), and arogenate dehydrogenase TyrA, at the last step in tyrosine biosynthesis (Figure 1A), revealed a differential pattern of metabolite accumulation in the pre‐chorismate shikimate pathway compared with the post‐chorismate pathway leading to Tyr and Phe production. Furthermore, plant growth at low concentrations of glyphosate showed an unexpected accumulation of arogenate, a pathway intermediated located downstream of the enzyme EPSP synthase, the target of glyphosate.

## RESULTS

### PEEK‐ZIC‐HILIC chromatography can separate the intermediates of AAA biosynthesis

ZIC‐HILIC column chemistry is a popular choice for resolving a diverse range of polar compounds by LC–MS (Buszewski & Noga, 2012; McCalley, 2017), performing particularly well for separating complex mixtures and phosphorylated compounds (Hosseinkhani et al., 2022). Given the presence of phosphorylated metabolites in the pre‐chorismate shikimate pathway (Figure 1A), we evaluated the suitability of ZIC‐HILIC to analyze AAA pathway intermediates by LC–MS by injecting authentic standards for the three AAAs and 11 of their biosynthetic precursors, corresponding to those commercially available plus arogenate, which was synthesized and purified as described in Materials and Methods. Analysis of these standards by ZIC‐HILIC in acetonitrile:water gradient with 5 mm acetic acid–ammonium acetate buffer at pH 4, a typical HILIC mobile phase, resulted in poor peak shape for some compounds. This issue particularly impacted those molecules having a phosphate functionality, like DAHP and shikimate 3‐phosphate, as phosphate groups are known to have a high tendency to interact with metal oxides from the column and LC–MS hardware (Heaton & McCalley, 2016; Wakamatsu et al., 2005).

To overcome this limitation, the ZIC‐HILIC column and the UHPLC capillaries were replaced by polyether ether ketone (PEEK)‐coated ZIC‐HILIC column and tubing, further complemented with the inclusion of 5 μm of methylene diphosphonic acid (i.e., medronic acid) in the mobile phase to saturate potential phosphate binding sites in the LC–MS hardware (Hsiao et al., 2018). This chromatographic setup successfully resolved all analyzed compounds when injected as a mix (Figure 1B). High sensitivity was observed for most compounds analyzed as pure standards, with limits of detection (LOD) as low as 0.01 pmol per injection, and limits of quantification (LOQ) between 0.05 and 0.33 pmol per injection, and a 2–10% relative standard deviation (RSV) between consecutive injections (Table S1; Figure S1). However, comparatively much lower sensitivities were observed for shikimate 3‐phosphate, chorismate, prephenate, and arogenate, with LODs of 2.4, 8, 22, and 2.5 pmol/injection, respectively (Table S1; Figure S1).

### Prephenate and arogenate are better detected as their adduct ions, phenylpyruvate and phenylalanine, respectively

**Figure 2:**
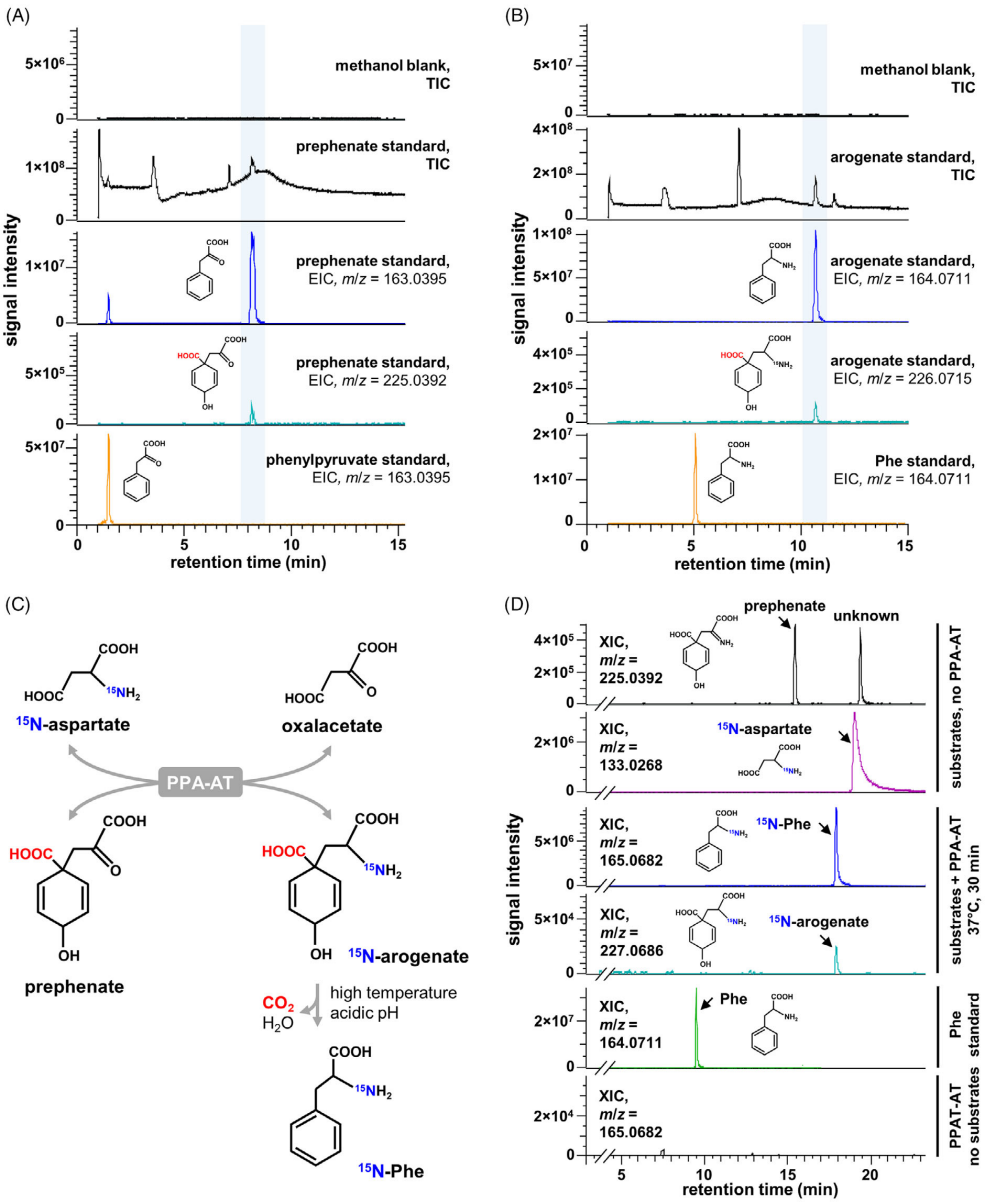
Prephenate and arogenate aromatize to phenylpyruvate and phenylalanine, respectively, during MS analysis. (A) Inspection of the total ion current (TIC) chromatogram of the prephenate standard in negative mode reveals a phenylpyruvate (*m*/*z* = 163.0395) ion at the same retention time as the prephenate ion (*m*/*z* = 225.0392), and different from the phenylpyruvate standard. (B) TIC in negative mode of the arogenate standard contains a phenylalanine (Phe; *m*/*z* = 164.0711) ion peak at the retention time of arogenate (*m*/*z* = 226.0715), and is different from the Phe standard. (C) Reaction scheme for the enzyme prephenate aminotransferase (PPA‐AT) catalyzing the conversion of prephenate and ^15^N‐labeled aspartate (^15^N‐aspartate) into ^15^N‐labeled arogenate (^15^N‐arogenate). (D) ^15^N‐arogenate (*m*/*z* = 227.0686) produced in the PPA‐AT reaction renders an overlapping ^15^N‐Phe (165.0682) peak at the same retention time. EIC, extracted ion chromatogram.

Prephenate and arogenate are chemically unstable compounds that undergo spontaneous decarboxylation and dehydration to phenylpyruvate and Phe, respectively, when exposed to acidic pH or high temperatures (Davis, 1953; Doy & Gibson, 1961; Jensen et al., 1977; Maeda et al., 2010; Metzenberg & Mitchell, 1956; Qian et al., 2019). As both conditions occur during LC–MS analysis, the low sensitivity in prephenate and arogenate detection (Figure S1) may be a consequence of their degradation. A careful inspection of the total ion current (TIC) trace for the prephenate standard showed a major peak corresponding with a phenylpyruvate ion in negative mode (*m*/*z* = 163.0395) overlapping the prephenate ion (*m*/*z* = 225.0392) at the same retention time (~8.3 min), which was different from that of a pure phenylpyruvate standard (~1.5 min) (Figure 2A). The phenylpyruvate ion overlapping the prephenate peak correlated with the amount of prephenate injected, but its signal was ~100‐times more intense than that of the prephenate ion, with an LOD around 50 times lower (22 pmol/injection for prephenate versus 0.44 pmol/injection for the phenylpyruvate adduct) (Figure 2A; Figure S2; Table S1). When the prephenate standard was treated with hydrochloric acid (HCl), which converts prephenate into phenylpyruvate (Davis, 1953; Doy & Gibson, 1961; Maeda et al., 2010; Qian et al., 2019), before injection, the phenylpyruvate ion at RT ~8.3 min and the prephenate ion became undetectable (Figure S3). Instead, a single phenylpyruvate peak appeared at the exact retention time of the pure phenylpyruvate standard, at ~1.5 min (Figure S3). Consequently, the phenylpyruvate ion that overlaps the prephenate peak is produced from prephenate degradation after LC separation, probably during MS analysis.

Similarly, the TIC trace for the arogenate standard showed a notable peak having the monoisotopic mass of Phe in negative mode (*m*/*z* = 164.0711) but at the same retention time (~10.7 min) as the expected arogenate ion peak (*m*/*z* = 226.0715) and clearly different from a Phe standard (~5.1 min) (Figure 2B). MS2 fragmentation data from an authentic Phe standard and the *m*/*z* ~164.7011 adduct of arogenate were collected and searched in the database mzCloud (https://www.mzcloud.org/), which found l‐Phe as the best candidate molecule in both cases (Figure S4). The intensity of this Phe adduct was positively correlated with that of the arogenate parent ion but had a much lower LOD than the arogenate ion (2.7 pmol/injection for the arogenate ion, versus 0.01 pmol/injection for the Phe adduct) (Figure 2B; Figure S2; Table S1). Incubating the arogenate standard in HCl before injection, which aromatizes arogenate into Phe (Jensen et al., 1977), made the arogenate and the arogenate‐overlapping Phe peaks undetectable, instead yielding a Phe peak at the retention time of the Phe standard (Figure S3). These observations support that like prephenate, arogenate largely converts to Phe during MS analysis.

To further confirm if the Phe ion at a retention time of ~10.7 min is the product of arogenate conversion, we used the enzyme prephenate aminotransferase (PPA‐AT) (Figure 1A) (Dal Cin et al., 2011; Graindorge et al., 2010; Maeda et al., 2011) to produce ^15^N‐labeled arogenate (^15^N‐arogenate) from prephenate using ^15^N‐labeled l‐aspartate as the amino donor of the transamination reaction (Figure 2C). LC–MS analysis of the PPA‐AT reaction products detected ^15^N‐Phe and ^15^N‐arogenate at the same retention time (Figure 2D), which was different from the retention time of an unlabeled Phe standard analyzed in the same run (Figure 2D). These findings support that the Phe ion peak that overlaps with the arogenate ion is a product of arogenate aromatization during the MS analysis. Therefore, this arogenate‐derived Phe ion and the analogous phenylpyruvate ion derived from prephenate can be used for highly sensitive and accurate quantification of arogenate and prephenate.

### Metabolite extraction at basic pH enables recovery of additional AAA biosynthetic intermediates

Common protocols for extracting plant metabolites are derived from the Bligh and Dyer method (Bligh & Dyer, 1959; Feussner & Feussner, 2019), in which plant samples are homogenized in a hydrophobic organic solvent (or a mixture of them) followed by a two‐phase separation after adding water to the homogenate. However, this general extraction approach is not well suited to extract key intermediates of AAA biosynthesis such as prephenate and arogenate, as they are highly unstable in moderately acidic aqueous solutions (Jensen et al., 1977; Maeda et al., 2010; Metzenberg & Mitchell, 1956; Qian et al., 2019). To overcome this limitation, we replaced the water added during the phase separation step with a solution of 1% 2‐amino‐2‐methyl‐1‐propanol (AMP) buffer adjusted to pH 10, which has been previously used for the extraction of intact prephenate and arogenate from plant samples (Maeda et al., 2010; Qian et al., 2019; Razal et al., 1994). Additionally, all extraction steps were performed on ice, and centrifugation steps at 4°C.

**Figure 3:**
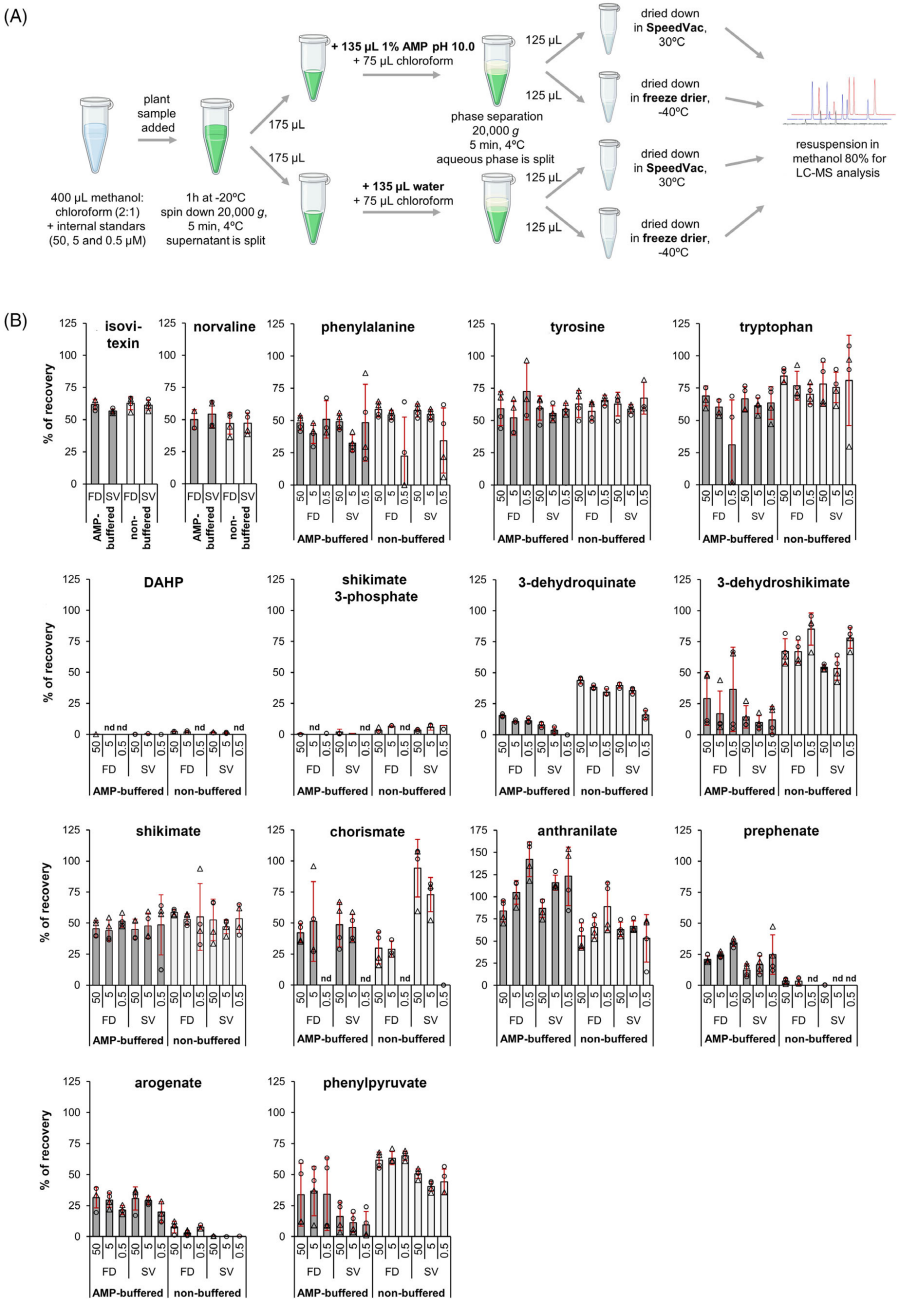
Extraction method comparison for the recovery of aromatic amino acids and their biosynthetic precursors from plant samples. (A) Schematic representation of the alternative extraction protocols, using AMP‐buffered or pure water (i.e., non‐buffered) for phase separation. (B) Compound recovery rate compared with the initial mass of compound spiked into the methanol: chloroform extraction solvent. Isovitexin and norvaline, two common internal standards used in plant metabolomics, were also included in the extraction solvent. Individual aromatic amino acids (phenylalanine, tryptophan and tyrosine) and AAA pathway metabolites were tested at three independent initial concentrations of 50, 5, and 0.5 μm, as indicated below each graph bar. Bars represent the average of *n* = 4 replicates coming from two independent recovery assays conducted on different days, each one consisting of *n* = 2 replicates, except for 3‐dehydroquinate, whose recovery was determined in a single experiment of *n* = 3. Individual datapoints coming from each one of these two independent experiments are indicated with circles or triangles. Error bars represent SD. AMP, 2‐amino‐2‐methyl‐1‐propanol buffer; FD, samples dried in a freeze‐dried; nd, not detected; SV, samples dried in a SpeedVac.

The suitability of this modified extraction method was first tested by spiking authentic standards for the compounds of interest at three different concentrations (0.5, 5, and 50 μm) into the starting methanol:chloroform solvent mixture, and determining their recovery rate compared with two reference internal standards, norvaline and isovitexin, in the presence of plant tissues, that is, leaves of 1‐month‐old Arabidopsis plants. The recovery rates of the AMP‐buffered extraction were also compared side‐by‐side with that of a regular extraction (i.e., non‐buffered) (Figure 3A). Furthermore, we tested the impact of drying the extracts in a SpeedVac at room temperature versus a freeze drier below −40°C (Figure 3A). The average recovery rates were determined (Table 1) based on four individual data points, from two independent extractions conducted on two different days (Figure 3B).

**Table 1:** Percentage recovery rate of the AAAs and their pathway intermediates following the AMP‐buffered and the non‐buffered methods compared with that of the internal standards norvaline and isovitexin

	Phe	Tyr	Trp	DAHP	DQNA	DSKA	SKA	SKAP	CHA	ATA	PPA	AGN	PPY
AMP‐buffered extraction													
Freeze drier	82 ± 18	107 ± 30	114 ± 14	0	7 ± 7	63 ± 41	84 ± 13	0	84 ± 39	196 ± 52	48 ± 11	50 ± 13	50 ± 45
SpeedVac	77 ± 32	105 ± 13	115 ± 16	0	17 ± 4	22 ± 18	85 ± 25	2 ± 2	85 ± 25	196 ± 45	32 ± 20	49 ± 14	22 ± 14
Non‐buffered extraction													
Freeze drier	89 ± 36	111 ± 15	140 ± 18	4 ± 2	42 ± 16	115 ± 9	100 ± 27	9 ± 2	53 ± 18	127 ± 42	7 ± 4	11 ± 7	133 ± 24
SpeedVac	91 ± 31	117 ± 15	140 ± 39	2 ± 2	48 ± 6	83 ± 15	94 ± 20	11 ± 4	156 ± 39	113 ± 30	0	0	115 ± 26

Compounds were spiked in the chloroform:methanol extraction solvent extract at three alternative concentrations of 50, 5, and 0.5 μm. The % recovery of the different metabolites was calculated based on the average recovery of the internal standards, isovitexin, and norvaline, which was set as 100% recovery. Values represent the average ± SD of *n* = 4 replicates from two independent of *n* = 2 technical replicates each and conducted on two different days, except for 3‐dehydroquinate (DQNA), whose recovery was determined based on a single experiment with *n* = 3. Original data are displayed in Figure 3(B).

AAA, aromatic amino acid; AGN, arogenate; AMP, 2‐amino‐2‐methyl‐1‐propanol; ATA, anthranilate; CHA, chorismate; DAHP, 3‐deoxy‐d‐*arabino‐*heptulosonate 7‐phosphate; DSKA, 3‐dehydroshikimate; DQNA, 3‐dehydroquinate; Phe, phenylalanine; PPA, prephenate; PPY, phenylpyruvate; SKA, shikimate; SKAP, shikimate 3‐phosphate; Trp, tryptophan; Tyr, tyrosine.

Overall, the three AAAs and shikimate were recovered with efficiencies comparable to that of norvaline and isovitexin, between 80 and 100%, without significant differences between extraction methods (Table 1; Figure 3B). The metabolites 3‐dehydroquinate, 3‐dehydroshikimate, and phenylpyruvate were better recovered in the non‐buffered extraction (Table 1; Figure 3B), which also performed better for anthranilate as the AMP‐buffered extraction seemingly overestimated its recovery (Figure 3B). In contrast, prephenate and arogenate could only be recovered in the AMP‐buffered extraction, although still at low relative recovery efficiency, ~35–50% compared with the internal standards (Table 1; Figure 3B). The phosphorylated intermediates DAHP and shikimate 3‐phosphate were barely recovered (<10%) (Table 1; Figure 3B). However, their recovery was similar to the internal standards when the extraction test was conducted without plant samples (Figure S5), suggesting that low recoveries are caused by plant matrix effects rather than degradation during the extraction. The recovery of chorismate was highly variable among replicates, and no significant recovery was obtained at the lowest concentration tested (0.5 μm) likely due to low sensitivity in chorismate detection (Table S1; Figure S1).

To better assess the method's sensitivity for plant samples, the LOD of the different compounds was determined in the presence of Arabidopsis leaf tissue spiking at different compound concentrations in the extractant solvent, between 0.5 and 5 μm (equal to nmol ml^−1^ of in the methanol:chloroform extractant). Based on previous findings (Figure 3B), 3‐dehydroquinate, 3‐dehydroshikimate, shikimate, anthranilate, Tyr and Trp, were analyzed following the non‐buffered method, whereas prephenate and arogenate were analyzed in AMP‐buffered extractions. Phe and phenylpyruvate were also determined in the AMP‐buffered extractions, as their quantification in non‐buffered extractions would likely overestimate them due to aromatization of the plant's internal pools of arogenate and prephenate, respectively. Freeze‐drying was used for all extractions, both non‐buffered and AMP‐buffered. DAHP, shikimate 3‐phosphate, and chorismate were not analyzed, either because of their very low recoveries (for DAHP and shikimate 3‐phosphate; Figure 3B) or poor detection sensitivity (for chorismate; Table S1).

Determination of LOD and LOQ using Arabidopsis leaf extracts as a matrix showed LODs between 0.01 and 0.05, and LOQs from 0.05 to 0.25 nmol ml^−1^ of extractant (Table S2) for all compounds but shikimate and prephenate, that exhibited LODs of 0.15 and 0.55, and LOQs of 0.73 and 2.75 nmol ml^−1^ of extract, respectively (Table S2). %RSV values were similar to those of the pure authentic standards (Table S1), in the range of 2–4% (Table S2). LOD and LOQ could not be determined for phenylpyruvate due to the very low signal‐to‐noise ratio and poor reproducibility between extractions, likely resulting from low phenylpyruvate stability at alkaline pH.

To evaluate if this analytical approach could be applied to plant samples other than Arabidopsis leaves, we conducted equivalent experiments using leaf and stem tissue from 6‐weeks‐old *N. benthamiana* plants. LOD, LOQ, and %RSV parameters were all together in a similar range compared with those observed in Arabidopsis leaf tissue, including comparatively lower sensitivities for shikimate and prephenate and highly inaccurate phenylpyruvate quantification (Table S2). Therefore, simultaneous extraction using both the AMP‐buffered and non‐buffered conditions can deliver an accurate quantification of the AAAs and many of their metabolic precursors in different plant tissues.

### AMP‐buffered extraction shows that Arabidopsis *sota* mutants hyperaccumulate arogenate and prephenate

The developed analytical pipeline was then used to determine the levels of AAAs and their biosynthetic precursors *in planta*. As a model of study, Arabidopsis *Col‐0* plants were compared with two *sota* mutant lines, *sotaA4* and *sotaB4*, which accumulate high levels of shikimate and AAAs as a result of mutations in the regulatory domain of the enzyme DAHP synthase (Figure 1A) (Yokoyama, de Oliveira, et al., 2022). Rosette leaves of 1‐month‐old Arabidopsis plants were extracted side‐by‐side following the AMP‐buffered and the non‐buffered extraction, both using a freeze drier (Figure 3A). To account for the low recovery of prephenate and arogenate compared with norvaline and other metabolites (Table 1; Figure 3B), ^15^N‐arogenate (Figure 2C) was included as an additional internal standard.

Five AAA pathway intermediates could be detected in Arabidopsis plants: 3‐dehydroshikimate, shikimate, prephenate, arogenate, and phenylpyruvate. Based on the findings from the recovery assays (Table 1; Figure 3B), endogenous levels of 3‐dehydroshikimate, shikimate, and phenylpyruvate were determined in the non‐buffered extracts, while prephenate and arogenate were quantified in the AMP‐buffered extracts. DAHP and shikimate‐3‐phosphate were not detected in plant extracts, whereas 3‐dehydroquinate, chorismate, and anthranilate peaks could not be unambiguously identified (Figure S6).

**Figure 4:**
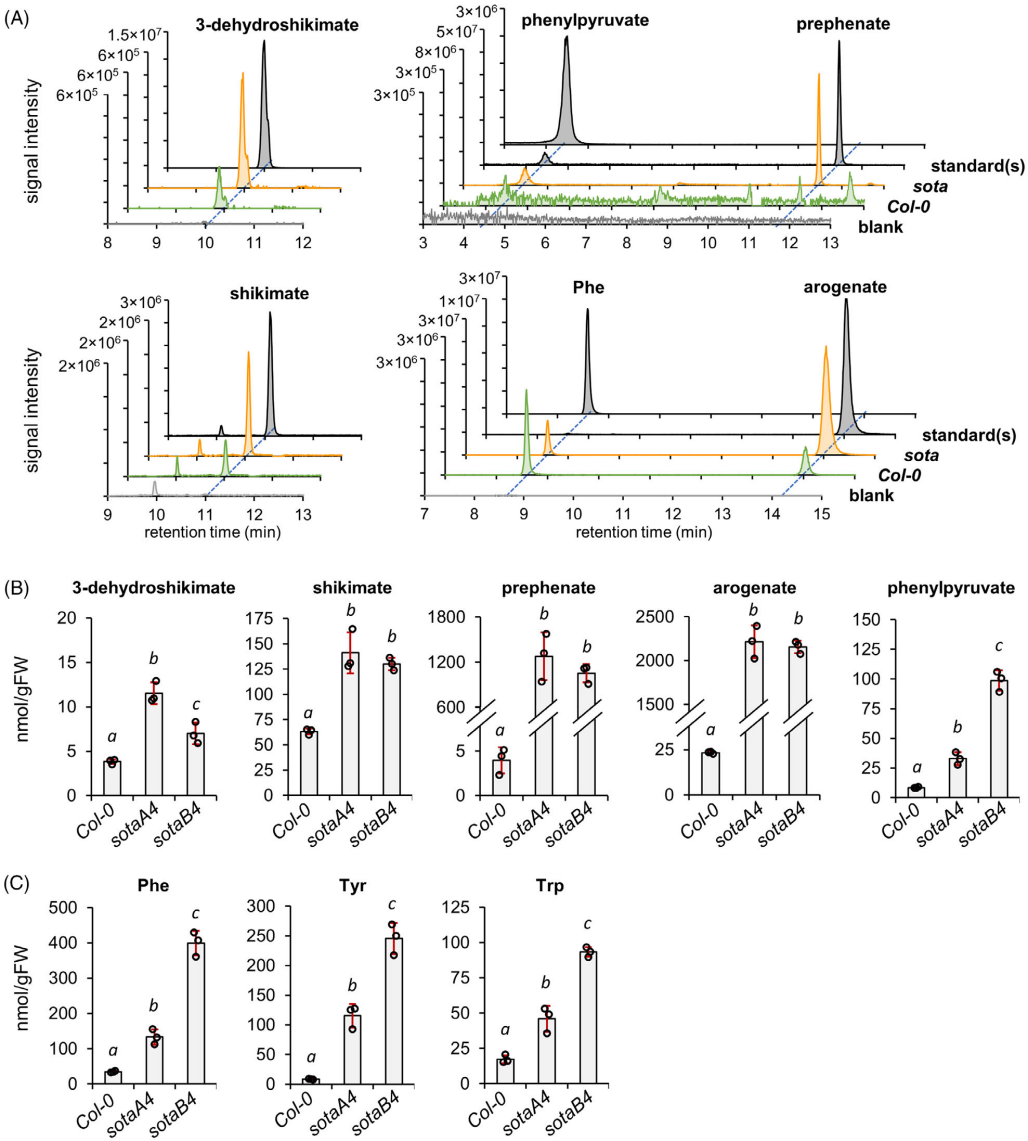
Quantification of aromatic amino acids and their pathway precursors in Arabidopsis rosette leaves. (A) Extracted ion chromatograms in negative mode for the aromatic amino acid pathway intermediates detected in Arabidopsis *Col‐0* and *sota* plants, compared with the methanol blank and the corresponding authentic standards injected at 25 pmol/injection. (B) Levels of aromatic amino acid pathway intermediates and (C) the three aromatic amino acids in Arabidopsis *Col‐0* and two *sota* mutant lines. 3‐dehydroshikimate, shikimate, phenylpyruvate, Tyr and Trp were determined in non‐buffered extracts, and their abundance was corrected by the recovery rate of the norvaline internal standard. Prephenate and arogenate were determined in the AMP‐buffered extracts, and their abundance corrected by the recovery rate of the ^15^N‐arogenate internal standard. Phe levels were also determined in AMP‐buffered extracts, but their abundance was calculated based on the recovery rate of the norvaline internal standard. Bars represent the average of *n* = 3 biological replicates coming from independent plants. Error bars = SD. Different letters indicate statistically significant differences for *α* = 0.05 according to Student's *t*‐test (two‐tailed test, equal variance).

Consistent with previous studies (Yokoyama, de Oliveira, et al., 2022; Yokoyama, Kleven, et al., 2022), the *sota* mutants accumulated higher levels of AAA and AAA precursors than *Col‐0* (Figure 4A). However, absolute abundance and fold change compared with *Col‐0* control varied substantially depending on the specific metabolite. The intermediates of the shikimate pathway, 3‐dehydroshikimate, and shikimate, increased by roughly two to three times in *sota* compared with *Col‐0*: ~4 versus ~12 nmol gFW^−1^ for 3‐dehydroshikimate, and ~60 versus ~140 nmol gFW^−1^ for shikimate, in *Col‐0* and *sota*, respectively (Figure 4B). Strikingly, the intermediates of the post‐chorismate pathway, prephenate, and arogenate, were drastically elevated by 250‐ and 100‐times, respectively, in *sota* plants compared with *Col‐0*: according to ^15^N‐arogenate corrected results, *sota* and Col had ~1000 versus 4 nmol gFW^−1^ for prephenate, while ~2000 versus 20 nmol gFW^−1^ for arogenate (Figure 4B). Phenylpyruvate increased from ~10 nmol gFW^−1^ in Col‐0 to 200–250 nmol gFW^−1^ in *sota* (Figure 4B), though a significant proportion of this phenylpyruvate might come from degradation of prephenate under acidic (i.e., non‐buffered) conditions. Therefore, *sota* plants accumulated high levels of the Phe and Tyr precursors arogenate and prephenate.

Regarding the AAAs, Tyr levels were ~7, 120, and 270 nmol gFW^−1^ in *Col‐0*, *sotaA4*, and *sotaB4*, respectively, whereas Trp levels were around 12, 40, and 90 nmol gFW^−1^, respectively (Figure 4C). Although Tyr and Trp levels were very similar in both extraction methods from plant tissue, Phe levels were up to six‐times higher in the non‐buffered extraction compared with the AMP‐buffered extraction (Figure S7). Quantification of ^15^N‐Phe, coming from the degradation of the ^15^N‐arogenate internal standard, revealed that a significant proportion (~50%) of the starting ^15^N‐arogenate had degraded into ^15^N‐Phe during the non‐buffered extraction (Figure S7). Conversely, when following the AMP‐buffered extraction, the net conversion of ^15^N‐arogenate to ^15^N‐Phe was minimal, between 1 and 5% (Figure S7). Hence, the conventional non‐buffered extraction method can substantially overestimate Phe content due to the degradation of the internal arogenate pool, specifically in samples with a large pool of arogenate.

### Coexpression of feedback‐insensitive DAHP synthase and TyrA enzymes in *N. benthamiana* had a different impact in the pre‐ and post‐chorismate pathways

**Figure 5:**
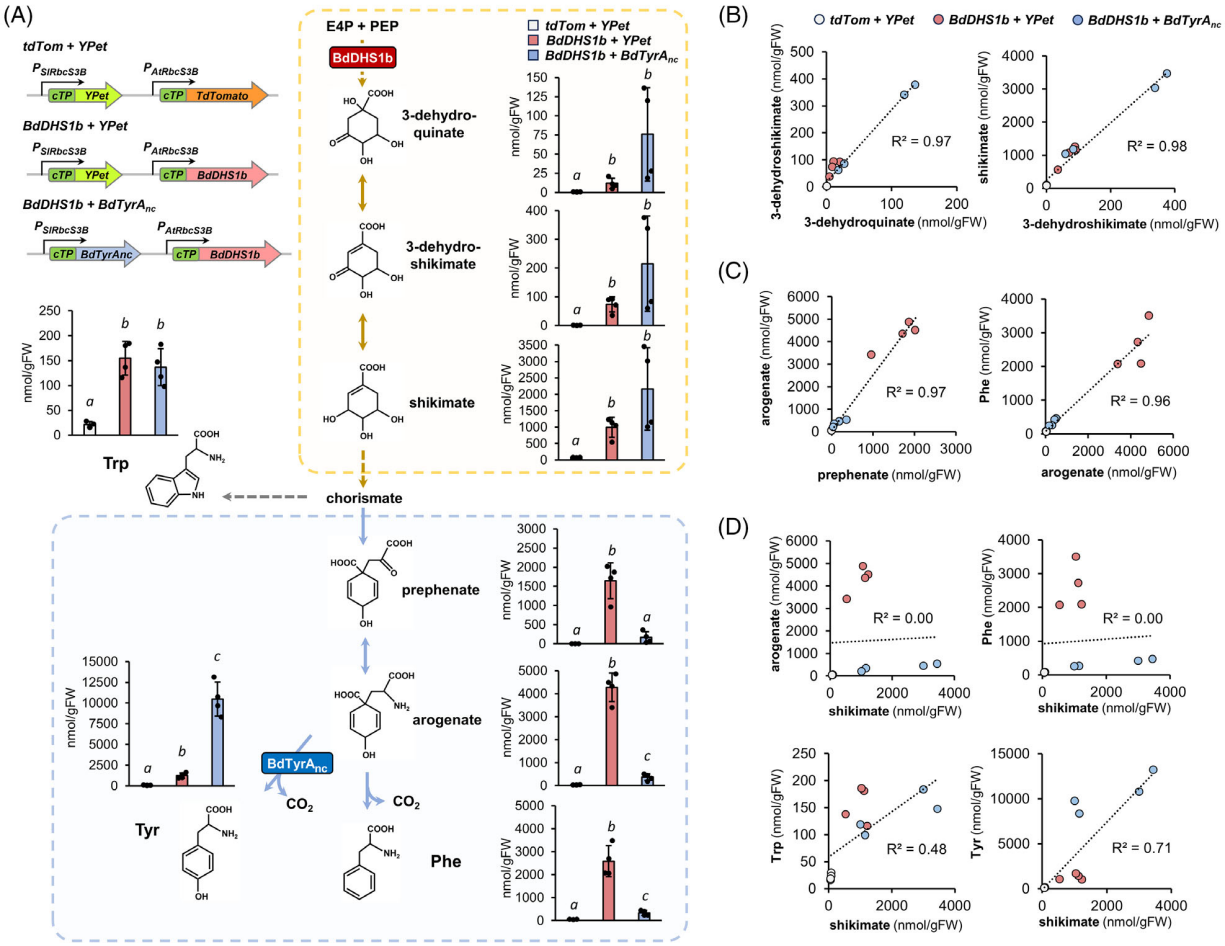
Quantification of aromatic amino acids and their pathway precursors in *Nicotiana benthamiana* leaves overexpressing the deregulated enzymes BdDHS1b and BdTyrA_nc_. (A) Levels of aromatic amino acids and their pathway intermediates. (B–D) Correlations between the levels of pre‐ and post‐chorismate pathway intermediates and aromatic amino acids, as quantified in (A). Figure legend as in (B). Metabolites were quantified in the water or AMP‐buffered extracts as described in the legend of Figure 4. Bars represent the average of *n* = 4 biological replicates coming from independent plants. Error bars = SD. Different letters indicate statistically significant differences for *α* = 0.05 according to Student's *t*‐test (two‐tailed test, equal variance).

To contrast the findings of DAHP synthase activity deregulation in Arabidopsis, we analyzed *N. benthamiana* leaves with and without expressing two feedback‐deregulated enzymes from *Brachypodium distachyon* that we described in a recent study (El‐Azaz et al., 2023): the DHAP synthase BdDHS1b, at the entry point of the shikimate pathway, and the arogenate dehydrogenase BdTyrA_nc_, at the last step in tyrosine biosynthesis (Figure 5A). *Agrobacterium*‐infiltrated leaves expressing *BdDHS1b* with and without *BdTyrA*_*nc*_, or the fluorescent proteins *TdTomato* and *YPet* as controls, were harvested at 3 days post‐infiltration and analyzed.

Besides 3‐dehydroshikimate and shikimate, the two intermediates of the shikimate pathway that could be quantified in Arabidopsis (Figure 4), 3‐dehydroquinate was also confidently detected and quantified in *N. benthamiana* samples (Figure 5). Upon *BdDHS1b* expression the levels of these three intermediates increased significantly: 3‐dehydroquinate raised from ~1 to ~12 nmol gFW^−1^ (~10‐times), 3‐dehydroshikimate from ~1.3 to ~70 nmol gFW^−1^ (~50‐times) and shikimate from ~80 to ~1000 nmol gFW^−1^ (~10‐times), in *TdTomato* + *Ypet* and *BdHDS1b* overexpressing leaves, respectively (Figure 5A). In the post‐chorismate arogenate pathway, prephenate increased from ~4 to ~1600 nmol gFW^−1^ (~400‐times), arogenate from ~40 to ~4300 nmol gFW^−1^ (~100‐times), Phe from ~60 to ~2600 nmol gFW^−1^ (~40‐times), and Tyr from ~100 to ~2000 nmol gFW^−1^ (~20‐times), in *tdTomato* + *Ypet* and *BdDHS1b* samples, respectively (Figure 5A). Trp levels increased from ~25 nmol gFW^−1^ in the *tdTomato* + *Ypet* control, to ~150 nmol gFW^−1^ in *BdDHS1b* samples (~6‐times) (Figure 5A). No other AAA pathway intermediate (DAHP, shikimate 3‐phosphate, chorismate, anthranilate) could be confidently detected in these samples. Similarly to what was observed in Arabidopsis *sota* mutants (Figure 4), the expression of a feedback‐deregulated DAHP synthase in *N. benthamiana* caused an overall increase in all detectable pathway intermediates, with arogenate becoming the most abundant one.

Compared with *BdDHS1b* expression alone, coexpression of *BdDHS1b* and *BdTyrA*_*nc*_ resulted in a marked reduction in the levels of prephenate and arogenate, while inducing the opposite trend in the pre‐chorismate shikimate pathway (Figure 5A). Among the AAAs, Tyr levels were increased to >10 000 nmol gFW^−1^, while Phe content was reduced by ~10‐times and Trp remained unchanged (Figure 5A). Interestingly, the levels of intermediates within the pre‐chorismate shikimate pathway (i.e., 3‐dehydroquinate, 3‐dehydroshikimate, shikimate) did not decrease ensuing *BdTyrA*_*nc*_ coexpression with *BdDHS1b* (Figure 5A). Correlation analyses among different metabolites showed positive correlation among 3‐dehydroquinate, 3‐dehydroshikimate, and shikimate across the different treatments (Figure 5B). Similarly, the intermediates in the post‐chorismate arogenate pathway (prephenate, arogenate) and Phe correlated positively (Figure 5C). However, shikimate levels (and by extension, 3‐dehydroquinate and 3‐dehydroshikimate) were poorly correlated with the levels of arogenate and AAAs (Figure 5D). Thus, our method could be successfully applied to *N. benthamiana*. Furthermore, the analysis revealed that pre‐ and post‐chorismate pathways respond differently upon genetic manipulation of DAHP synthase and TyrA enzymes.

### Metabolic profiling of plants growth at low concentrations of glyphosate reveals unexpected accumulation of arogenate

**Figure 6:**
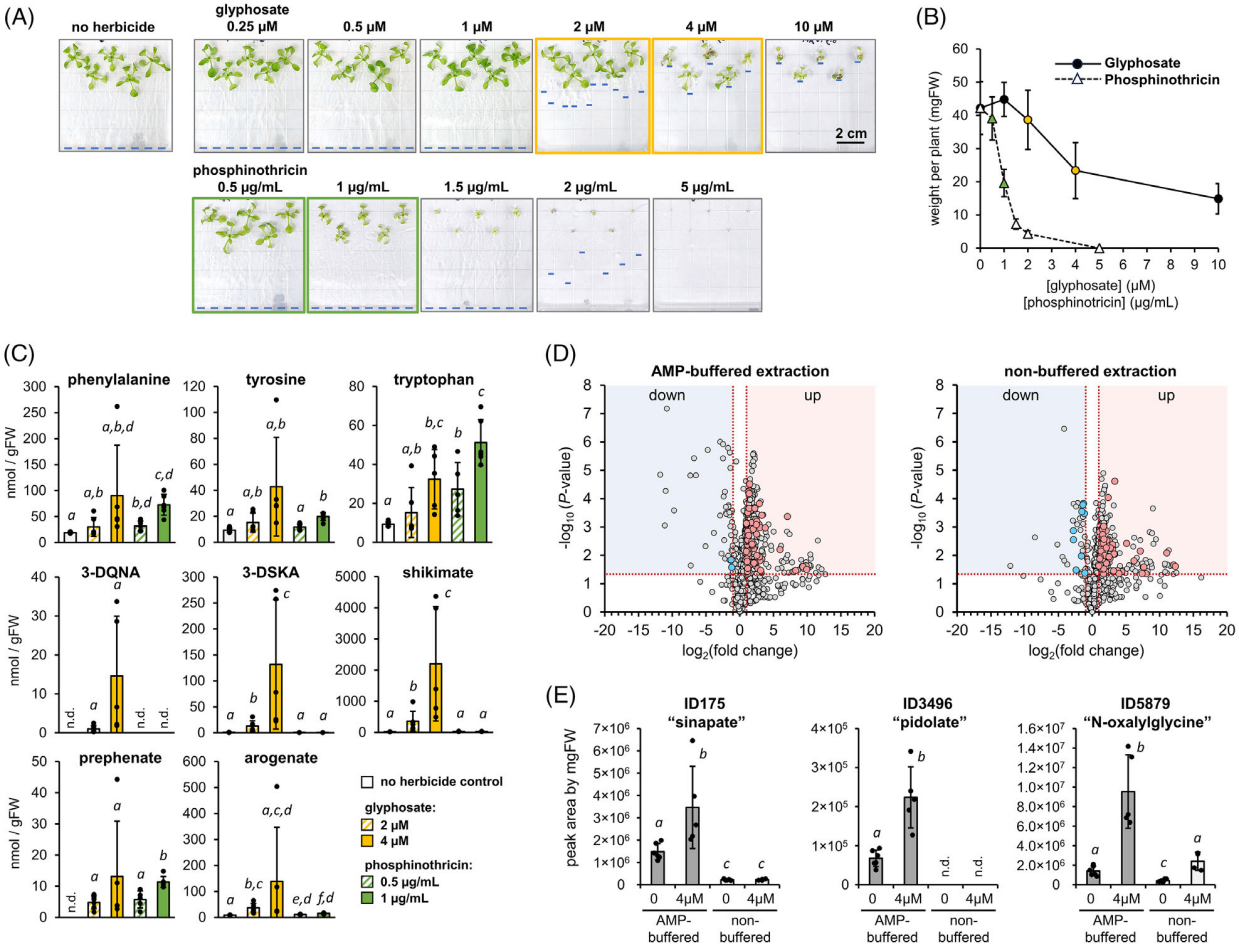
Targeted and untargeted metabolomics of Arabidopsis grown at low levels of the herbicides glyphosate and phosphinothricin. (A) Arabidopsis *Col*‐*0* plants were grown on half MS media plates supplemented with different levels glyphosate, an inhibitor of EPSP synthase in the shikimate pathway, or phosphinothricin, an inhibitor of nitrogen assimilation that targets glutamine synthetase. Blue lines on the plates mark the length of the roots. (B) Fresh weight (in mg) of the rosettes of 15‐day‐old plants grown at different concentrations of glyphosate or phosphinothricin. Colored dots correspond to herbicide doses causing approximately a 10% and 50% reduction in rosette total mass. (C) Levels of AAAs and their pathway intermediates in plants grown at different doses of glyphosate or phosphinothricin. Metabolites were quantified in the water or AMP‐buffered extracts as described in the legend of Figure 4. Bars represent the average of *n* = 5–6 biological replicates coming from independent plants. Error bars = SD. Different letters indicate statistically significant differences for *α* = 0.05 according to Student's *t*‐test (two‐tailed test, equal variance). (D) Volcano plots comparing the metabolome of plants grown at 4 μm glyphosate control plates, depending on the extraction method used. Horizontal dashed lines correspond to a *P* < 0.05 according to Student's *t*‐test (two‐tailed test, equal variance). Vertical lines correspond to a fold change of ≥2 times. Colored dots represent features that in addition to changing significantly upon glyphosate treatment, were also detected at ≥3‐times higher intensity in the corresponding extraction method compared with the other. (E) Manual integration of three mass features significantly accumulated upon glyphosate treatment that was better measured in the AMP‐buffered extraction compared with the non‐buffered extraction. Bars represent the average of *n* = 4–5 biological replicates coming from independent plants. Error bars = SD. Different letters indicate statistically significant differences for *α* = 0.05 according to Student's *t*‐test (two‐tailed test, equal variance).

We next evaluated the effect of constraining the carbon flow through the shikimate pathway by the action of the herbicide glyphosate, which inhibits the shikimate pathway enzyme EPSP synthase (Figure 1A) (Steinrücken & Amrhein, 1980). Arabidopsis *Col*‐0 plants were grown for 15 days on Murashige‐Skoog (MS) media plates supplemented with a range of low doses of glyphosate between 0 and 10 μm (Figure 6A). To distinguish the effects specific to glyphosate from those potentially related to a general disruption in amino acid homeostasis, plants were also grown at different concentrations of another common herbicide, phosphinothricin, an inhibitor of the enzyme glutamine synthetase (Gill & Eisenberg, 2001). After evaluating the impact of herbicide dose on plant growth (Figures 5A and 6B), we analyzed two alternative concentrations for each herbicide, causing a ~10% and ~50% reduction in the fresh weight of the rosette (Figure 6B). These concentrations corresponded to 2 and 4 μm for glyphosate, and 0.5 and 1 μg ml^−1^ for phosphinothricin (Figure 6B).

Metabolite profiling in the control and herbicide plates showed a general tendency toward increased levels of AAAs in response to both glyphosate and phosphinothricin (Figure 6C; Figure S8), as previously reported in Arabidopsis (Trenkamp et al., 2009). Significant differences between glyphosate and phosphinothricin treatments were observed for other proteinogenic amino acids, such as glutamate, proline, or the nitrogen‐dense amino acids glutamine, arginine, and asparagine (Figure S8).

As expected, glyphosate, unlike phosphinothricin, caused a dose‐dependent increase in the levels of 3‐dehydroquinate (otherwise not detectable in Arabidopsis control plants), 3‐dehydroshikimate and shikimate (Figure 6C), which are all located upstream of the EPSP synthase step (Figure 1A). The increase in 3‐dehydroshikimate and shikimate levels at 4 μm glyphosate was on average >100‐times compared with the control plates (Figure 6B), consistent with previous reports (Mueller et al., 2003; Trenkamp et al., 2009; Zabalza et al., 2017; Zulet‐Gonzalez et al., 2023). Surprisingly, glyphosate treatment also increased the levels of prephenate and arogenate (Figure 6C), which are located downstream of EPSP synthase. Specifically, arogenate levels increased from ~10 nmol gFW^−1^ in the control plates to ~30 nmol gFW^−1^ at 2 μm glyphosate, with some of the plants grown at 4 μm glyphosate – particularly, those individuals experiencing a more severe reduction in growth, as shown in Figure 6A – having as much as ~500 nmol gFW^−1^ of arogenate (Figure 6C). Although similar prephenate accumulation was also triggered by phosphinothricin (Figure 6C), the high accumulation of arogenate was unique to glyphosate.

### Extraction of plant metabolites at alternative pH can increase the power of untargeted metabolomic approaches

To explore the applicability of the AMP‐buffered extraction besides the AAA pathway, we performed an untargeted metabolomics analysis using the data from plants grown in control and 4 μm glyphosate plates (Figure 6A). Full‐range negative mode MS1 data from the AMP‐buffered and non‐buffered extractions coming from the same individual plants were analyzed in MZmine (Schmid et al., 2023). Associated MS2 data were used to predict compound identity using SIRIUS (Dührkop et al., 2019). After the removal of ions present in the blanks and filtering out potentially redundant features, including ^13^C‐isotopes, the untargeted workflow identified a total of 1112 mass features present in at least 25% of the plant samples analyzed and having a signal intensity above 2.0 × 10^5^, that is, >20‐times the typical signal noise observed during the run (Data S1).

To identify compounds that may be better quantified using AMP‐buffered extraction at basic pH, we selected the mass features being ≥3 times more abundant in the AMP‐buffered extractions compared with the conventional non‐buffered extraction, which resulted in around 200 features (~18% of the total) (Data S1). These ~200 features were further restricted to those changed by ≥2 times either up or down in the 4 μm glyphosate plates compared with the control plates (Figure 6D), resulting in 111 features that changed upon glyphosate treatment (109 became more abundant, and 2 less abundant) and were better or exclusively detected in the AMP‐buffered extractions (Figure 6D; Data S1). In comparison, 60 features altered by the glyphosate treatment (51 up and 9 down) were better detected in the non‐buffered extractions, including two mass features predicted to be 3‐dehydroquinate and 3‐dehydroshikimate (Figure 6D; Data S1), as previously detected in our foregoing targeted analysis (Figure 6C).

To validate these results, we then performed a manual integration of 25 of the AMP buffer‐enriched and glyphosate‐changed features, selected based on having the best MS2‐based prediction structure score in SIRIUS and not sharing correlation group or retention times. We confirmed that 22 out of these 25 features were detected at a much higher intensity, if not exclusively, in AMP‐buffered than in non‐buffered extractions (Figure S9). Among them, we found compounds having a high structural similarity to the lignin pathway intermediate sinapate, the glutathione‐related metabolite pidolate, and the amino acid derivative N‐oxalylglycine, all three significantly upregulated in response to glyphosate (Figure 3A). Therefore, although their chemical identities need to be further verified, the use of alternative pH conditions during the extraction can increase the power of metabolomic approaches by expanding the number of detectable metabolites besides AAA pathway intermediates.

## DISCUSSION

Quantitative metabolite profiling of the shikimate and AAA pathways typically requires specific, sometimes indirect, and time‐consuming methods that may not be compatible with the simultaneous quantification of any other plant metabolites. Unlike in microbial cells (Aristilde et al., 2017; Lu et al., 2010; Oldiges et al., 2004), reports from plant tissues are scarce except for shikimate (Amrhein et al., 1980; Bochkov et al., 2012; Ding et al., 2007; García‐Romero et al., 1993; Qian et al., 2019; Tzin et al., 2012; Yokoyama, de Oliveira, et al., 2022; Yokoyama, Kleven, et al., 2022), and separated methods are required to measure shikimate, prephenate, arogenate, phenylpyruvate, and AAAs (Maeda et al., 2010, 2011; Qian et al., 2019). Often, detection of some targeted intermediates was only possible upon genetic modification (Ding et al., 2007; Shi et al., 2022), glyphosate application (Zabalza et al., 2017; Zulet‐Gonzalez et al., 2023), or the use of highly specific methods targeting each individual compounds. Here, we report an extraction procedure and LC–MS analytical approach for the simultaneous and accurate quantification of up to five AAA pathway intermediates in plant samples – 3‐dehydroquinate, 3‐dehydroshikimate, shikimate, prephenate, and arogenate – a significant improvement over previous studies. The method, and particularly the dichotomic metabolite extraction at alternative pH values (Figure 3A) also enables improved detection of many other plant metabolites beyond AAA pathway intermediates and products (Figure 6D,E; Data S1).

Detection of the upper shikimate pathway intermediates 3‐dehydroquinate and 3‐dehydroshikimate in wild‐type plants show remarkable low levels for these compounds (<5 nmol gFW^−1^; Figures 4B, 5A, and 6C) compared with shikimate and other downstream metabolites. However, 3‐dehydroquinate and 3‐dehydroshikimate accumulated upon DAHP synthase deregulation (Figures 4B and 5A) and glyphosate treatment (Figure 6C), possibly because of shikimate accumulation and the reversibility of the bifunctional DQD/SDH enzyme (Figure 1A) (Ding et al., 2007; Fiedler & Schultz, 1986; Maeda & Dudareva, 2012; Singh & Christendat, 2006), which may also explain why these compounds correlated positively (Figure 5B). Shikimate accumulation suggests that its further conversion by shikimate kinase (SK) and/or additional downstream enzyme(s) becomes rate‐limiting upon increasing carbon flux through the shikimate pathway by deregulating DAHP synthase activity.

Prephenate and arogenate, precursors to Phe and Tyr biosynthesis (Figure 1A), are highly labile at acidic pH (Davis, 1953; Doy & Gibson, 1961; Jensen et al., 1977; Metzenberg & Mitchell, 1956), and thus their quantification requires specific extraction methods at alkaline pH followed by derivatization (Maeda et al., 2010; Razal et al., 1994; Tzin et al., 2012, 2013), or alternatively, indirect quantitative approaches via conversion into phenylpyruvate and Phe, respectively (Maeda et al., 2010, 2011; Qian et al., 2019; Yoo et al., 2013). Prephenate and arogenate could be readily detected in our plant samples using the AMP‐buffered extraction (Figures 4, 5, 6). Although we included an ^15^N‐arogenate internal standard to determine accurate absolute quantification data, this additional internal standard is not essential to make relative comparisons in prephenate and arogenate content between control and treated samples, as both compounds recover at low but similar levels through independent extractions using the AMP‐buffered method (Figure 3B). Comparison of Phe and arogenate levels between AMP‐buffered and non‐buffered extracts support that many previous Phe reports in plant tissues are potentially overestimated due to extraction under non‐buffered conditions that cause the aromatization of the arogenate pool.

Consistent with our observations (Figures 4 and 5), previous studies in Arabidopsis and *N. benthamiana* reported drastic increases in AAAs levels upon increasing carbon flow through the shikimate pathway by deregulating its first step, catalyzed by DAHP synthase (El‐Azaz et al., 2023; Tzin et al., 2012, 2013; Yokoyama, Kleven, et al., 2022; Yokoyama, de Oliveira, et al., 2022). Here we found that these plant samples had more than 100 times the levels of prephenate and arogenate than the corresponding controls (Figures 4 and 5), which was not detected in previous studies that used conventional (i.e., non‐buffered) metabolite extraction (El‐Azaz et al., 2023; Yokoyama, Kleven, et al., 2022; Yokoyama, de Oliveira, et al., 2022). The hyper‐accumulation of prephenate and arogenate indicates that the conversion of arogenate to Phe and Tyr by arogenate dehydratase (ADT; EC 4.2.1.91) and TyrA enzymes, respectively (Figure 1A), became limiting upon the expression of deregulated DAHP synthases. Consistently, the coexpression of *BdTyrAnc* with *BdDHS1b* alleviated this bottleneck for Tyr production, substantially reducing arogenate levels (Figure 5). Interestingly, we found that the intermediates of the pre‐chorismate shikimate pathway remained unchanged or even at higher levels compared with *BdDHS1b* plants alone (Figure 5), suggesting the existence of additional and distinct regulation mechanisms between the upstream shikimate pathway and the downstream post‐chorismate branches for AAA production.

Limiting carbon flow through the shikimate pathway by growing Arabidopsis plants at low doses of the EPSP synthase inhibitor glyphosate (Steinrücken & Amrhein, 1980) caused high increases in shikimate levels at its precursors 3‐dehydroshikimate and 3‐dehydroquinate, the latter becoming detectable in Arabidopsis after this treatment. This finding is consistent with the inhibition of EPSP synthase, and has been observed before in different plant species treated with glyphosate (Mueller et al., 2003; Trenkamp et al., 2009; Zabalza et al., 2017; Zulet‐Gonzalez et al., 2023). However, we also detected an unexpected accumulation of prephenate and arogenate, two metabolites located downstream of EPSP synthase. Although the increase in prephenate levels was relatively low and was also observed upon phosphinothricin treatment (Figure 6C), the increase in arogenate content was more intense and seemingly specific to glyphosate but not phosphinothricin treatment (Figure 6C). A similarly paradoxical accumulation of arogenate has also been observed in *Petunia* × *hybrida* plants upon silencing the gene encoding the enzyme PPA‐AT, which converts prephenate into arogenate (Figure 1A) (Maeda et al., 2011). It remains to be determined whether the observed arogenate accumulation is caused by decreased consumption upon downregulation of TyrA or ADT enzymes, or instead by increased arogenate production through an unknown mechanism.

Although this analytical approach is a significant improvement compared with previous methods, it is not free of limitations. The phosphorylated precursors DAHP and shikimate 3‐phosphate (Figure 1A) could be efficiently recovered in a mock extraction without plant tissue (Figure S5) but not when plant tissue was added to the extractant (Figure 3B). Chorismate detection was overall very poor even when injecting the authentic standard (Figure S1; Table S1). Efficient and accurate extraction of phenylpyruvate also remains challenging, as this compound is underestimated at alkaline pH due to decreased stability and recovery (Figure 3; Table 1), whereas at acidic pH prephenate will aromatize and cause phenylpyruvate overestimation. In contrast, the absence of anthranilate detection suggests very low *in planta* levels, as our quantification method is highly sensitive for this compound (Tables S1 and S2). Besides these areas for future improvement, this study showed that extraction of plant metabolites at alternative pH conditions can increase the power of untargeted metabolomic approaches beyond AAA pathway products and intermediates. Detailed analysis of multiple intermediates using the analysis developed here revealed that the plant shikimate and post‐chorismate AAA pathways are two distinct and independently regulated functional modules. This finding has a broad implication toward a more effective bioengineering of plant AAA and phenolic metabolism.

## EXPERIMENTAL PROCEDURES

### Plant materials and growth conditions

Arabidopsis seeds from *Col*‐0, *sotaA4* and *sotaB4* were directly sown in 7 × 7 cm pots filled with Jiffy's Soilless Mix substrate and stratified for 2 days at ~5°C in the dark. After stratification, plants were kept for a month in an environmental chamber at 22°C, 60% relative humidity, 12 h photoperiod under ~100 μE of light intensity. Plants were regularly watered with a 1:10 dilution of Hoagland's solution.

For the herbicides treatment, Arabidopsis *Col*‐0 seeds were chlorine‐sterilized and sown on half‐strength MS plates with 0.3% gelrite and 0.5% sucrose supplemented with different concentrations of glyphosate and phosphinothricin (see Figure 6A). The sown plates were stratified at ~5°C for 3 days and then transferred to the same growth conditions stated in the previous paragraph, where they were kept for 15 days. The harvesting (whole plant rosette without roots) was conducted at ~8 h after the beginning of the light period by flash freezing. The samples were kept at −80°C until analysis.

*Nicotiana benthamiana* plants used for transient expression experiments were germinated on Jiffy's Soilless Mix substrate for ~10 days at 22°C in a 12 h‐photoperiod under ~200 μE of light intensity, covered by a transparent plastic dome. After germination, seedlings (approx. 1 cm wide) were transplanted into 10 × 10 cm pots filled with Jiffy's Soilless Mix substrate and grown for 3 weeks at 22°C in a 12 h‐photoperiod, ~200 μE of light intensity, 60% humidity. Plants were regularly watered with a 12:4:8 (N:P:K) plant nutritive solution (Miracle‐Gro) at a 1:1000 dilution.

### Metabolite extraction from plant samples

For metabolite recovery determination (Table 1; Figure 3B), authentic standards of the compounds of interest were individually spiked at three different concentrations of 0.5, 5 and 50 μm within 400 μl of cold chloroform:methanol mix (1:2), together with 25 μm of norvaline and 0.5 μg ml^−1^ of isovitexin as internal standards. Around 30–40 mg of pulverized frozen plant tissue from Col‐0 Arabidopsis leaves were then resuspended into the chloroform:methanol mix, kept on ice for ~1 h with regular vortexing, followed by centrifugation at 20 000 ***g*** for 5 min at 4°C. After centrifugation, the supernantant was split into two fresh tubes at 175 μl each, and mixed with 135 μl of 1% AMP buffer pH 10 (for AMP‐buffered extracts) or 135 μl of LC–MS grade water (for non‐buffered extracts), plus 75 μl of chloroform. The samples were then vortexed vigorously for 5 min, spun down at 20 000 ***g*** for 5 min at 4 °C, and the aqueous supernatant split again into two fresh tubes at 125 μl each, which were dried down overnight in a SpeedVac at ~30°C, or a freeze drier at ~−40°C. The dried pellets were resuspended into 100 μl of methanol 80% with 1 mm NaOH for AMP‐buffered extracts, or 100 μl of methanol 80% for non‐buffered extracts, sonicated in a water bath for 5 min, and then spun down at 20 000 ***g*** for 5 min at 4°C. The supernatant was transferred to LC vials for injection.

For *in planta* determination of metabolite levels (Figures 4 and 5), 30–40 mg of pulverized frozen leaves were resuspended into 400 μl of freshly prepared cold chloroform:methanol (1:2) with 25 μm of norvaline, 0.5 μg ml^−1^ of isovitexin, and 10 μm of ^15^N‐arogenate as internal standards. Extracts were kept for ~1 h on ice with regular vortexing, followed by centrifugation at 20 000 ***g*** for 5 min at 4 °C. The supernantant was split into two fresh tubes at 175 μl each and then mixed with 135 μl of 1% AMP buffer pH 10 (for AMP‐buffered extracts) or 135 μl of LC–MS grade water (for non‐buffered extracts), plus 75 μl of chloroform. After 5 min of vigorous vortexing, the tubes were spun down at 20 000 ***g*** for 5 min at 4°C, and 250 μl of the aqueous supernatant was transferred to a fresh tube to be dried down overnight in a freeze drier. The dried pellets were resuspended into 100 μl of methanol 80% with 1 mm NaOH for AMP‐buffered extracts, or 100 μl of methanol 80% for non‐buffered extracts, sonicated in a water bath for 5 min, and then spun down at 20 000 ***g*** for 5 min at 4°C. The supernatant was transferred to LC vials for injection.

### ZIC‐HILIC‐MS analysis and quantification of plant metabolites

AAAs and their pathway precursors were detected using a Vanquish Horizon Binary UHPLC (Thermo Scientific, Waltham, MA, USA) coupled to a Q Exactive Orbitrap mass spectrometer (Thermo Scientific, Waltham, MA, USA). One microliter of the sample was analyzed using a InfinityLab Poroshell 120 HILIC‐Z PEEK‐lined column (150 × 2.1 mm, 2.7‐μm particle size; Agilent) kept at 30°C. Agilent's stainless steel PEEK‐lined PK/ST RLO/RLO BIO capillaries (⌀ 0.17 mm) were used to connect the column to the UHPLC and MS. Solvent A was made of 5 mm ammonium acetate/0.2% acetic acid buffer in water supplemented with 5 μm (final concentration in A) of methylene diphosphonic acid. Solvent B was composed of 5 mm ammonium acetate/0.2% acetic acid buffer in 95% acetonitrile. The following gradient, expressed as %B in v/v, was used for all analysis, with the exception of the results shown in Figure 2D and Figure S3: 0–2 min, 100%; 2–8 min, 100–85%; 8–15.75 min, 85–75%; 15.75–16 min, 75–20%; 16–18 min, 20%; 18–18.25 min, 20–100%; 18.25–22 min, 100%. The flow rate was 0.45 ml min^−1^. For the gradient used on Figure 2D and Figure S3, see Table S3. All chemicals used to prepare the mobile phases were LC–MS grade.

MS data were collected using both full scan and SIM in negative mode between 1 and 16 min, under the following parameters: sheath gas flow rate, 55; auxiliary gas flow rate, 20; sweep gas flow rate, 2; spray voltage, 3 kV; capillary temperature, 350°C; S‐lens RF level, 50; resolution, 70 000; AGC target 3 × 106, maximum scan time 100 msec; scan range 70–1050 m/z. Spectral data were integrated manually using Xcalibur 3.0 according to the parameters shown in Table S4.

Compound identity and abundance were determined based on calibration curves made using high‐purity authentic standards (Figure S1) and divided by the mass of fresh tissue extracted (in g of fresh weight) and the recovery factor of the internal standard. Quantified internal pools of all metabolites from the plant tissue, except prephenate and arogenate, were adjusted based on the average recovery rate of the internal standards isovitexin and norvaline (typically 50–60%, see Figure 3B). Prephenate and arogenate were instead normalized based on the recovery of the ^15^N‐arogenate internal standard. Correction of metabolite abundance by that of the corresponding internal standard was performed according to the following formula:

$$\text{Compound abundance}=\frac{\text{compound peak area}\;}{\text{internal standard recovery factor}}$$

where the internal standard recovery factor, from 0 to 1, was calculated dividing the peak area of the internal standard(s) in each sample by the total peak area of the same internal standard, determined as the average of three independent buffer blanks. The buffer blanks were prepared by directly drying down 400 μl (the same volume used for plant tissue extraction) of the exact same methanol:chloroform extractant solvent used for the plant tissue extraction for 10 min in a SpeedVac, therefore having 25 μm norvaline, 0.5 μg μl^−1^ isovitexin and 10 μm ^15^N‐arogenate, and resuspending the pellet in a final volume of 100 μl of methanol 80% (the same final resuspension volume used for the plant extracts). Equal recovery was assumed regardless of the concentration of the metabolite in the plant sample.

### Determination of LOD, LOQ, and %RSV

LOD and LOQ were defined as the concentration of compound giving a signal‐to‐noise ratio of at least two‐ and ten‐times, respectively, upon injection of 1 μl of sample. %RSV corresponds to the relative standard deviation, expressed as % of the average, of five consecutive injections of 1 μl of the same sample having around the calculated LOQ of the compound.

For the pure authentic standards, LOD and LOQ were determined using the lowest detectable concentration of the standard curve (Figure S1; Table S1). For LOD and LOQ determination in plant tissues (i.e. Arabidopsis leaves, and *N. benthamiana* leaves and stems), six alternative concentrations of compound (0.5, 1, 2, 3, 4, and 5 μm, in addition to a no‐compound control having only plant tissue) were spiked into 400 μl of the methanol:chloroform extraction solvent, and ~30 mgFW of the corresponding ground frozen plant tissue were added. The samples were then incubated on ice for ~1 h with regular vortexing, spun down at 20 000 ***g*** for 5 min at 4°C, and the whole supernantant (~400 μl) was transferred to a fresh tube, where 300 μl of either 0.5% AMP buffer pH 10 (for Phe, prephenate, arogenate and phenylpyruvate) or LCMS‐grade water (for the rest of compounds; see Table S2). The extractions were then vortexed vigorously for 5 min and spun down at 20 000 ***g*** for 5 min at 4°C. The aqueous fraction was then recovered almost completely (~500 μl) without disturbing the hydrophobic interphase and dried down overnight in a freeze drier at ~−40°C. Resuspension of the dried pellets was conducted as described in previous sections (see “Metabolite extraction from plant samples” section). LOD and LOQ were determined in these samples by measuring the signal‐to‐noise ratio of the quantified ion at the two lowest detectable concentrations tested. The peak area of the compound was subtracted when the same ion could be detected in the no‐compound control having only plant tissue. %RSV was determined in five consecutive injections of 1 μl of the same sample having around the calculated LOQ of the compound.

### Untargeted metabolomics of glyphosate‐treated Arabidopsis plants

High‐throughput integration was conducted in MZmine v4.1.0 (Schmid et al., 2023) using full‐range TIC (*m*/*z* 90 to 900) negative polarity data collected between 1 and 20 min for the AMP‐buffered and non‐buffered extracts of 15‐days‐old plants grown in half MS media plates without (*n* = 6 individuals) or with 4 μm of glyphosate (*n* = 5 individuals) and four extraction mocks for identifying and removing contaminant features. The whole rosette of each individual plant, without roots, was extracted and analyzed individually. Extraction mocks were made by following the exact same extraction protocol described for the AMP‐buffered method, but without adding any plant sample to the extraction.

For feature detection, the noise threshold was set to 1.0 × 10^4^ and 2.0 × 10^3^ for MS1 and MS2, respectively. Chromatograms were built with the LC–MS chromatogram builder tool for mass features with a minimum absolute height of ≥2.0 × 10^5^ and detected in at least seven consecutive scans, with a minimum intensity of 1.0 × 10^5^ between peaks. Local minimum resolver was applied for a minimum ratio of peak top/edge of three and a maximum peak duration of 1 min. Carbon‐13 isotopes were then removed using the ^13^C isotope filter tool. Features were aligned with the join aligner tool using a retention time and *m*/*z* tolerance of 0.2 min and 10 ppm, respectively. Redundant features were consolidated using the duplicate feature filter. Features present in at least two out of four mock extractions were subtracted from the feature list, except for features at least three times more abundant in the plant samples compared with the extraction mock, which were kept. Gaps in the blank‐subtracted feature list were filled using the feature finder tool. After gap‐filling, features not being present in at least five plant samples were removed using the feature list rows filter tool, and a correlation analysis of the remaining features was performed in metaCorrelate. The final features list was then exported as both molecular networking files and SIRIUS outputs with merged MS2 spectra. The exported feature list was further analyzed in MS excel, the integrated peak area divided by the mass of the plant sample (in mgFW), and the recovery factor of isovitexin (determined in a manual integration) to perform statistical analysis across the dataset.

The SIRIUS output file was used to predict feature identity and structure in SIRIUS v5.8.6 (Dührkop et al., 2019), allowing [M − H]− and [M + Cl]− as possible ionizations. ZODIAC (Ludwig et al., 2020) was enabled at default parameters to improve the search. A structure search was performed with the CSI:FingerID (Dührkop et al., 2015) in all available databases.

### Transient expression in *N. benthamiana*

Construct generation and transient expression of *BdDHS1b* and *BdTyrA*_*nc*_ in the leaves of *N. benthamiana* were performed as described in El‐Azaz et al. (2023). Briefly, *BdDHS1b* and *BdTyrA*_*nc*_ genes were cloned from leaf cDNA of *B. distachyon* (genotype Bd21) into Golden Gate level 0 vectors (Engler et al., 2014; Weber et al., 2011) and assembled into a Golden Gate level 2 binary backbone (pAGM4673; Weber et al., 2011) for simultaneous expression. The vector backbone also contained an expression cassette for the gene‐silencing repressor *p19*, which was found critical for *BdDHS1b* expression in *N. benthamiana* (El‐Azaz et al., 2023). *Agrobacterium tumefaciens* strain GV3101 transformed with the plant expression constructs were grown at 28°C for 24–36 h in 10 ml of LB liquid media containing the corresponding antibiotics. The saturated cultures were spun down at 3000 ***g*** for 5 min at room temperature, washed twice with 3 ml of induction media (IM; 10 mm MES [2‐(*N*‐morpholino)ethanesulfonic acid] buffer pH 5.6, 0.5% glucose, 2 mm NaH_2_PO_4_, 20 mm NH_4_Cl, 1 mm MgSO_4_, 2 mm KCl, 0.1 mm CaCl_2_, 0.01 mm FeSO_4_, and 0.2 mm acetosyringone), incubated in 3 ml of IM for 2–3 h at room temperature in the dark, pelleted at 3000 ***g*** for 5 min and resuspended into 3 ml of 10 mm MES buffer pH 5.6 with 0.2 mm acetosyringone. OD_600nm_ was adjusted to a final density of 0.5 units using 10 mm MES buffer pH 5.6 with 0.2 mm acetosyringone. *N. benthamiana* plants of around 4‐week‐old were infiltrated close to the end of the light period into two different leaves per plant, following a randomized scheme. Samples consisting of the infiltrated leaf limbs, without the main veins, were harvested at ~72 h post‐infiltration and subjected to LC–MS metabolite analysis (see details below).

### Arogenate synthesis

Arogenate was synthesized enzymatically from prephenate (Millipore Sigma) and aspartate using purified recombinant PPA‐AT enzyme from Arabidopsis (Maeda et al., 2011) as previously described (Rippert & Matringe, 2002), and purified by ion exchange chromatography using a FPLC (Connelly & Siehl, 1987; Maeda et al., 2010). For the production of ^15^N‐arogenate, regular aspartate was replaced by ^15^N‐aspartate. Purity level and concentration of the arogenate preparations were determined by LC–MS before and after conversion of arogenate to Phe by acid treatment (Jensen et al., 1977) and subsequent quantification of Phe compared with an authentic standard.

### Statistics and reproducibility

All experiments shown in the manuscript were conducted independently at least twice to confirm the reproducibility of the findings, except for the determination of 3‐dehydroquinate recovery, which was based on a single experiment of *n* = 3. Statistical comparisons between samples (Student's *t*‐test) were performed using Microsoft Office Excel. No statistical method was used to predetermine the sample size. All the experiments conducted with plants were randomized. The Investigators were not blinded to allocation during experiments and outcome assessment.

## AUTHOR CONTRIBUTIONS

JEA and HAM conceived and designed the experiments. JEA performed the experiments and data analysis. The manuscript was drafted by JEA and revised by HAM.

## CONFLICT OF INTEREST

HAM and JEA have a pending patent application related to the naturally deregulated BdDHS1b enzyme described by El‐Azaz et al. (2023). The authors declare that they have no other competing interests.

## Supporting information


**Table S1.** Limit of detection, limit of quantification, and relative standard deviation between consecutive injections in pure authentic standards.
**Table S2.** Limit of detection, limit of quantification, and relative standard deviation between consecutive injections in plant extracts.
**Table S3.** Alternative UHPLC gradient used for the results shown in Figure 2(D) and Figure S3.
**Table S4.** Monoisotopic mass, ion mass, and retention times used for LCMS quantification of plant metabolites.
**Figure S1.** Calibration curves for the AAAs and their biosynthetic intermediates using PEEK‐ZIC‐HILIC.
**Figure S2.** Correlation between prephenate and arogenate ions with the overlapping phenylpyruvate and Phe ions, respectively.
**Figure S3.** LC–MS analysis of prephenate and arogenate standards before and after incubation in HCl.
**Figure S4.** MS2 fragmentation data supports that arogenate is transformed to a l‐phenylalanine adduct during LC–MS analysis.
**Figure S5.** Recovery assays for AAA pathway precursors in the absence of added plant material.
**Figure S6.** Extracted ion chromatograms corresponding to chorismate, anthranilate, and 3‐dehydroquinate ions in Arabidopsis rosette leaves.
**Figure S7.** Quantification of Phe and arogenate in non‐buffered versus AMP‐buffered extracts.
**Figure S8.** Determination of non‐aromatic free amino acid levels in Arabidopsis growth at low doses of glyphosate of phosphinothricin.
**Figure S9.** Manual integration of a selection of mass features enriched in the AMP‐buffered extraction compared with the non‐buffered extraction method.

**Data S1.** Untargeted metabolomic analysis of glyphosate‐treated plants.
