# Supplementary material for: A simplified liquid chromatography‐mass spectrometry methodology to probe the shikimate and aromatic amino acid biosynthetic pathways in plants
Source: Plant J. 2024 Oct 28;120(5):2286–304. doi: 10.1111/tpj.17105 (PMC11629745; doi:10.1111/tpj.17105)
Supplement: Supplementary file 1 — Table S1. Limit of detection, limit of quantification, and relative standard deviation between consecutive injections in pure authentic standards. Table S2. Limit of detection, limit of quantification, and relative standard deviation between consecutive injections in plant extracts. Table S3. Alternative UHPLC gradient used for the results shown in Figure 2(D) and Figure S3. Table S4. Monoisotopic mass, ion mass, and retention times used for LCMS quantification of plant metabolites. Figure S1. Calibration curves for the AAAs and their biosynthetic intermediates using PEEK‐ZIC‐HILIC. Figure S2. Correlation between prephenate and arogenate ions with the overlapping phenylpyruvate and Phe ions, respectively. Figure S3. LC–MS analysis of prephenate and arogenate standards before and after incubation in HCl. Figure S4. MS2 fragmentation data supports that arogenate is transformed to a l‐phenylalanine adduct during LC–MS analysis. Figure S5. Recovery assays for AAA pathway precursors in the absence of added plant material. Figure S6. Extracted ion chromatograms corresponding to chorismate, anthranilate, and 3‐dehydroquinate ions in Arabidopsis rosette leaves. Figure S7. Quantification of Phe and arogenate in non‐buffered versus AMP‐buffered extracts. Figure S8. Determination of non‐aromatic free amino acid levels in Arabidopsis growth at low doses of glyphosate of phosphinothricin. Figure S9. Manual integration of a selection of mass features enriched in the AMP‐buffered extraction compared with the non‐buffered extraction method. [file TPJ-120-2286-s001.pdf]

SUPPLEMENTAL MATERIAL FOR

A Simplified LC-MS Methodology to Probe the Shikimate and Aromatic Amino Acid Biosynthetic Pathways in Plants

El-Azaz and Maeda,

2024

Table S1. Limit of detection (LOD), limit of quantification (LOQ) and relative standard deviation between consecutive injections (%RSD) in pure authentic standards. n.d. = not determined.

	LOD (pmol/injection)	LOQ (pmol/injection)	%RSD
phenylalanine	0.01	0.07	2.1
tyrosine	0.07	0.33	5.2
tryptophan	0.04	0.19	7.0
DAHP	0.30	1.48	4.6
3-dehydroquinate	0.05	0.24	3.2
3-dehydroshikimate	0.02	0.08	1.8
shikimate	0.23	1.13	3.9
shikimate-3-phosphate	2.4	11.8	5.1
chorismate	8.0	40	3.1
anthranilate	0.01	0.05	3.0
prephenate (m/z 225.0399)	22	111	n.d.
prephenate (m/z 163.0395 adduct)	0.44	2.18	4.4
arogenate (m/z 226.0715)	2.7	13.3	n.d.
arogenate (m/z 164.0711 adduct)	0.01	0.05	2.1
phenylpyruvate	0.02	0.08	4.0

Table S2. Limit of detection (LOD), limit of quantification (LOQ) and relative standard deviation between consecutive injections (%RSD) in different plant tissues.

	Extraction method	LOD (nmol/mL)			LOQ (nmol/mL)			%RSD		
		At, leaves	Nb, leaves	Nb, stems	At, leaves	Nb, leaves	Nb, stems	At, leaves	Nb, leaves	Nb, stems
tyrosine	non-buffered, FD	0.05	0.04	0.07	0.23	0.21	0.34	2.6	2.4	2.9
tryptophan	non-buffered, FD	0.02	0.03	0.04	0.10	0.13	0.19	2.3	2.3	2.6
shikimate	non-buffered, FD	0.15	0.13	0.07	0.73	0.63	0.34	3.2	2.3	3.0
3-dehydroquinate	non-buffered, FD	0.03	0.03	0.03	0.13	0.15	0.13	2.2	2.7	1.8
3-dehydroshikimate	non-buffered, FD	0.01	0.02	0.01	0.05	0.10	0.05	1.6	2.1	2.2
anthranilate	non-buffered, FD	0.01	0.01	0.04	0.05	0.05	0.19	2.7	2.8	3.1
phenylalanine	AMP-buffered, FD	0.03	0.02	0.01	0.17	0.09	0.05	2.3	2.4	2.2
prephenate (*m/z* 163.0395 adduct)	AMP-buffered, FD	0.55	0.51	0.43	2.75	2.55	2.14	3.9	4.2	3.8
arogenate (*m/z* 164.0711 adduct)	AMP-buffered, FD	0.03	0.02	0.01	0.13	0.09	0.06	2.2	2.3	2.4

Table S3. Alternative gradient used for the results shown in Main Figure 2d and Figure S3. Mobile phase composition and chromatographic setup were as described in the main materials and methods section.

time (min)	Flow (mL/min)	%B
0.0	0.45	100
2.0	0.45	100
11.0	0.45	89
33.0	0.45	50
33.5	0.45	20
35.5	0.45	20
36.0	0.45	100
40.0	0.45	100

Table S4. Monoisotopic mass, ion mass and retention times used for LC-MS quantification of plant metabolites.

	Monoisotopic mass	Ionization mode	Quantified adduct	*m*/*z*	*m*/*z* window (ppm)	rt window (min)
Phenylalanine	165.0789	neg	$[M-H]^-$	164.0711	5	8.0 - 9.0
Tyrosine	181.0738	neg	$[M-H]^-$	180.0660	5	9.5 - 10.5
Tryptophan	204.0898	neg	$[M-H]^-$	203.0820	5	8.5 - 9.5
DAHP	288.0246	neg	$[M-H]^-$	287.0168	5	18.0 - 20.0
3-dehydroquinate	190.0477	neg	$[M-H]^-$	189.0399	5	9.5 - 11.0
3-dehydroshikimate	172.0371	neg	$[M-H]^-$	171.0293	5	9.5 - 10.5
Shikimate	174.0528	neg	$[M-H]^-$	173.0450	5	10.5 - 11.5
Shikimate-3-phosphate	254.0191	neg	$[M-H]^-$	253.0113	5	17.0 - 19.0
Chorismate	226.0477	neg	$[M-H]^-$	225.0399	5	14.5 - 15.5
Anthranilate	137.0476	neg	$[M-H]^-$	136.0398	5	1.0 - 1.5
Prephenate***	164.0473	neg	$[M-CO_2-H_2O-H]^-$	163.0395	5	11.0 - 12.0
Arogenate***	165.0789	neg	$[M-CO_2-H_2O-H]^-$	164.0711	5	13.0 - 14.5
Phenylpyruvate	164.0473	neg	$[M-H]^-$	163.0395	5	3.5 - 5.5

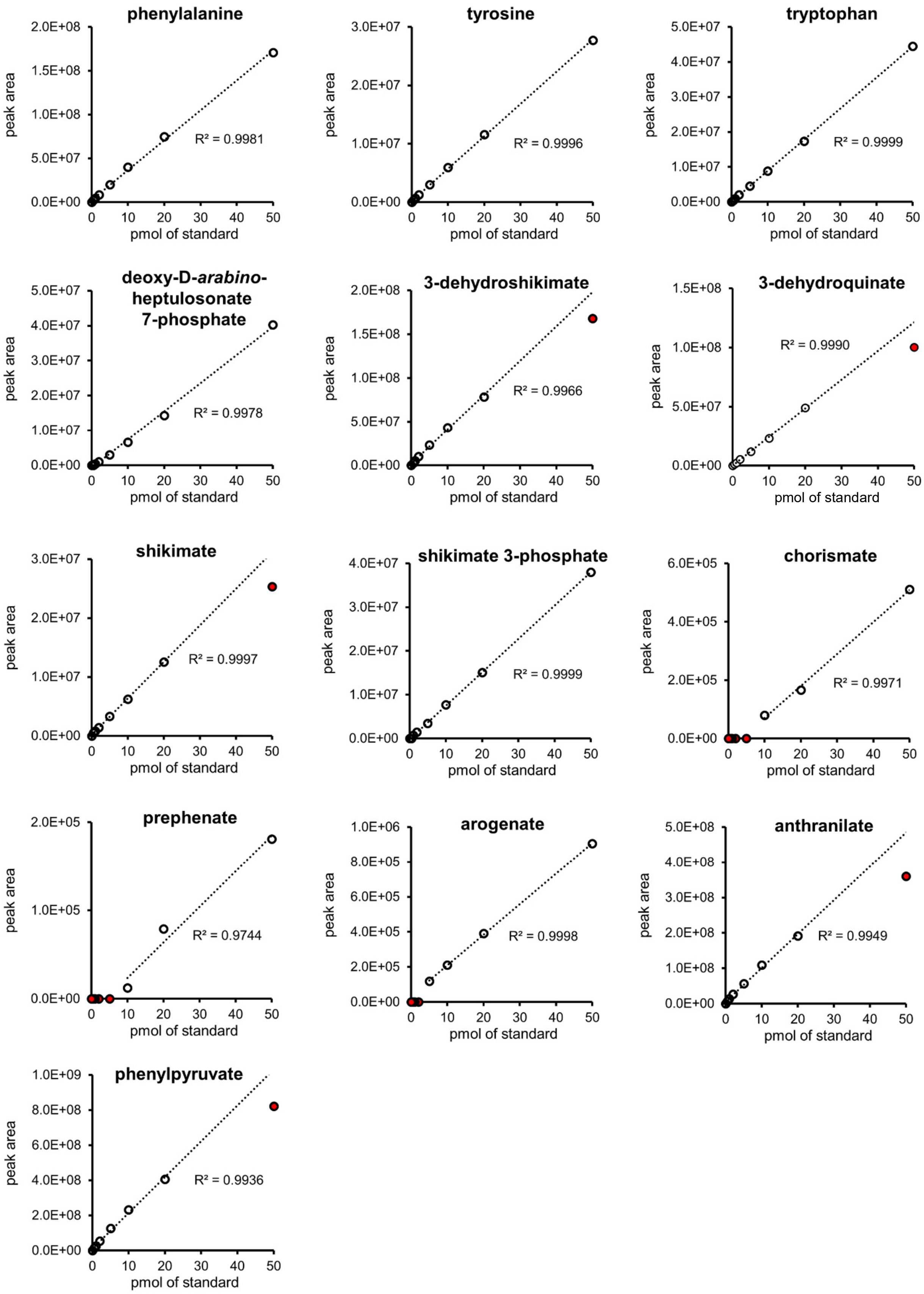
phenylalanine
R² = 0.9981
tyrosine
R² = 0.9996
tryptophan
R² = 0.9999
deoxy-D-*arabino*-heptulosonate 7-phosphate
R² = 0.9978
3-dehydroshikimate
R² = 0.9966
3-dehydroquinate
R² = 0.9990
shikimate
R² = 0.9997
shikimate 3-phosphate
R² = 0.9999
chorismate
R² = 0.9971
prephenate
R² = 0.9744
arogenate
R² = 0.9998
anthranilate
R² = 0.9949
phenylpyruvate
R² = 0.9936
peak area
pmol of standard

Figure S1 (previous page). Calibration curves for the AAAs and their biosynthetic intermediates using PEEK-ZIC-HILIC. Standards for all compounds were injected at 0.5, 1, 2, 5, 10, 20 and 50 pmol per injection (1 µL injection volume). Individual points correspond to a single injection. Dots marked in red were excluded from the linear regression analysis, as they were either not detected (chorismate, arogenate, prephenate) or saturating (3-dehydroshikimate, 3-dehydroquinate, shikimate, anthranilate, phenylpyruvate). Dashed lines correspond to the linear regression analysis of all datapoints, except those datapoints marked in red. R^2 = coefficient of determination from the linear regression analysis.

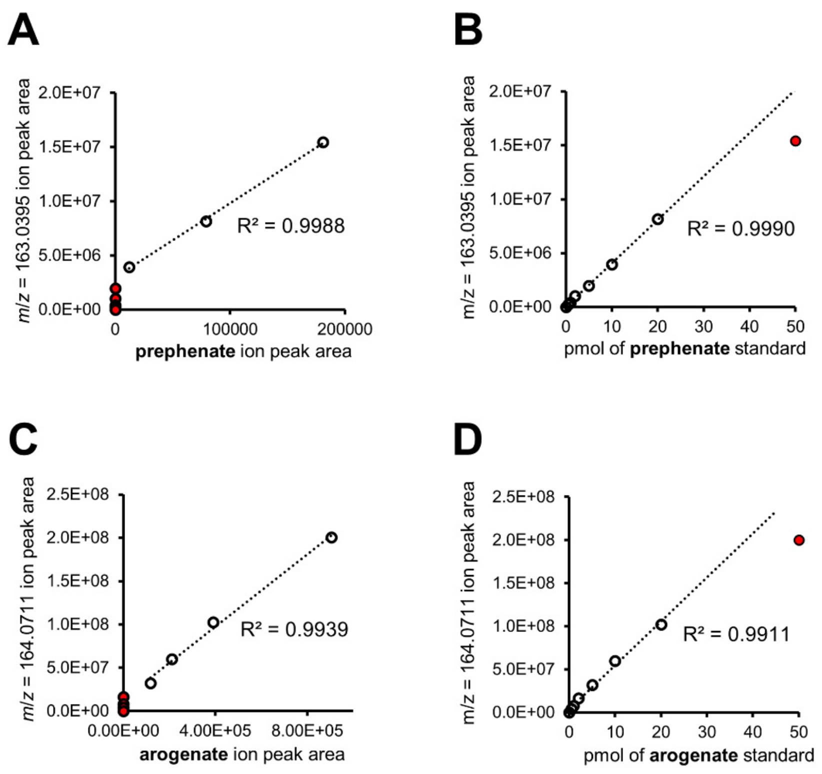

Figure S2. Prephenate and arogenate ion signal in the standard curves correlate with the overlapping phenylpyruvate and Phe ions, respectively. A) Correlation between the peak area of the prephenate ion and the peak area of the overlapping phenylpyruvate ion in negative mode (*m*/*z* = 163.0395). **B)** Correlation between the mass of prephenate standard injected and the peak area of the overlapping phenylpyruvate ion (*m*/*z* = 163.0395). **C)** Correlation between the peak area of the arogenate ion and the overlapping Phe ion in negative mode (*m*/*z* = 164.0711). **D)** Correlation between the mass of arogenate standard injected and the peak area of the overlapping Phe ion (*m*/*z* = 163.0395). Individual points correspond to a single analysis. Dots marked in red correspond to: in panels A and C, concentrations of prephenate or arogenate at which the expected prephenate or arogenate ion was undetectable; in panel B and D, saturated peak areas of the overlapping phenylpyruvate or Phe ions. Dashed lines correspond to the linear regression analysis of all datapoints, except those marked in red. R^2 = coefficient of determination from the linear regression analysis.

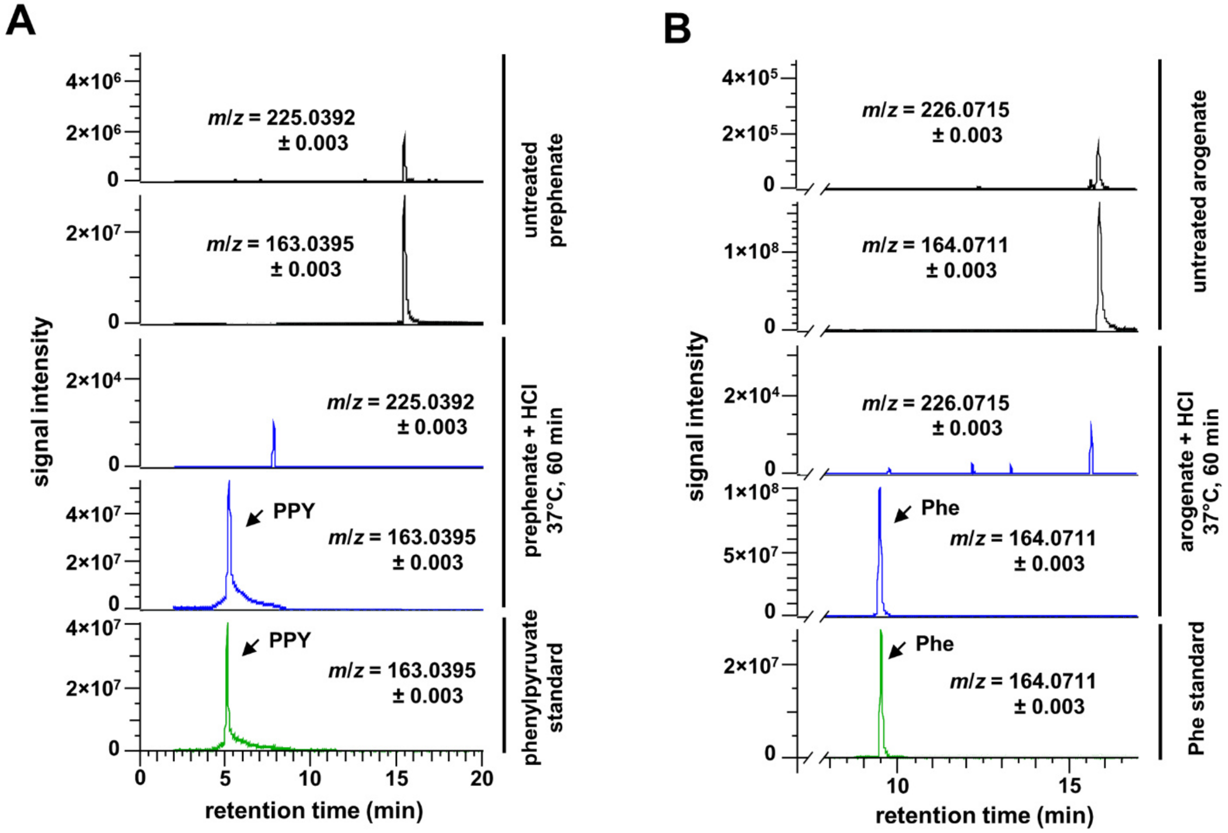

Figure S3. Prephenate and arogenate treated with HCl before LCMS analysis do not overlap with the phenylpyruvate (PPY) or phenylalanine (Phe) ion peaks. A) Prephenate standard overlaps with a phenylpyruvate ion (*m/z* = 163.0395). This phenylpyruvate peak becomes undetectable at the expected retention time (~16 min) if the prephenate standard is incubated with HCl, which causes the conversion of prephenate into phenylpyruvate, before LCMS analysis. **B)** Arogenate standard overlaps with a Phe ion (*m/z* = 164.0711) that becomes undetectable at the expected retention time (~17 min) if the arogenate standard is treated with HCl, and therefore converted to Phe (retention time ~9 min), before LCMS analysis. Note that the LC-MS gradient used for this experiment corresponds to Table S1, and is different from the gradient used for analysis of plant metabolite extracts (which is described in the main materials and methods section).

A

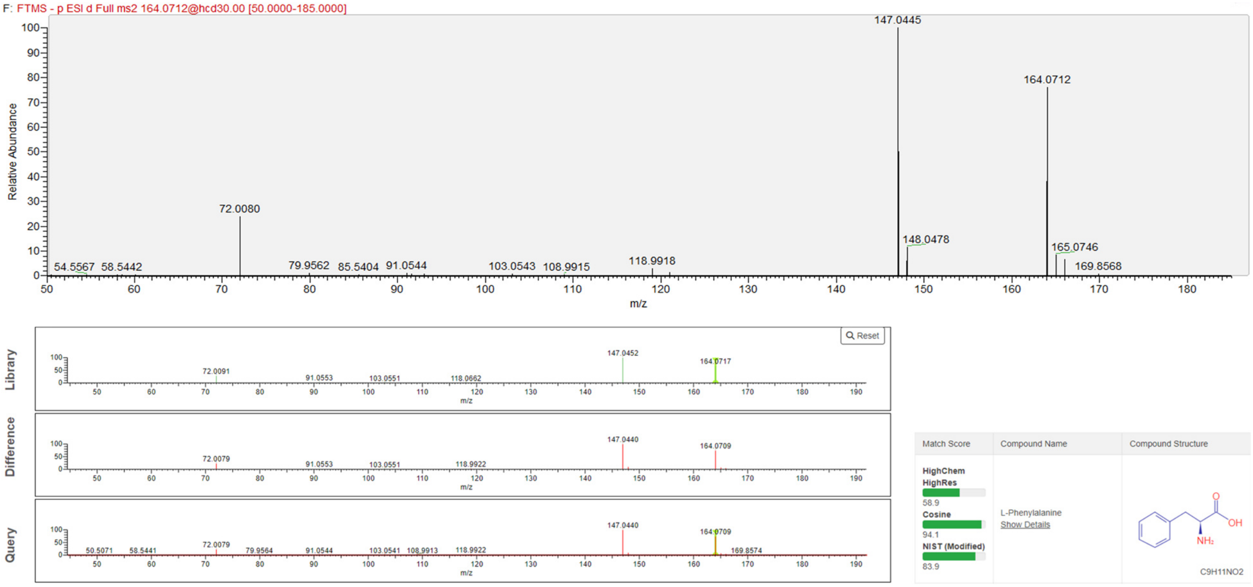

B

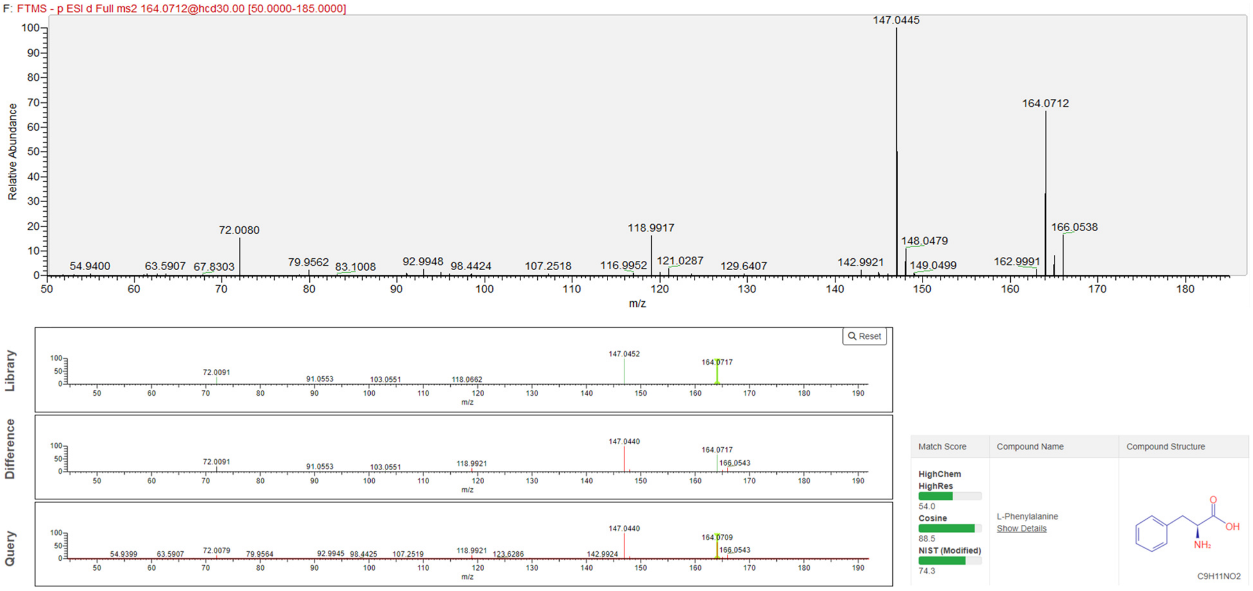

Figure S4. MS2 fragmentation data supports that arogenate is transformed to an L-phenylalanine adduct during LC-MS analysis. A) MS2 spectra of the *m/z* 164.0711 adduct of arogenate found in the arogenate standard at retention time ~ 14.0 and **B)** MS2 spectra from the [M-H]- phenylalanine ion from a L-phenylalanine standard (same m/z but different retention time). Both spectra were searched against the database mzCloud (https://www.mzcloud.org/) using identity search under default settings, which returned L-phenylalanine as the most likely candidate molecule in both cases.

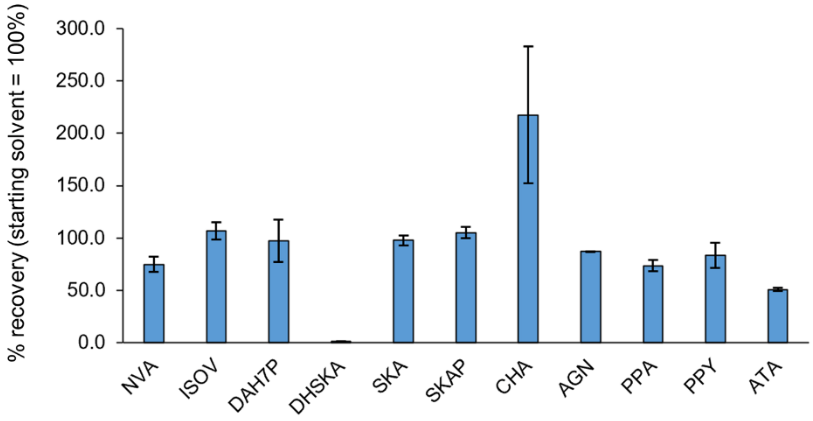

Figure S5. Recovery assay using internal standards spiked into the extraction solvent, with no plant sample added, following the AMP-buffered extraction protocol. The stability of the compounds in the during the extraction procedure was tested with no interference of plant materials/other chemicals. Compound abbreviation: NVA, norvaline; ISOV, isovitexin; DAH7P, 3-deoxy arabino 7-phosphate; DHSKA, 3-dehydroshikimate; SKA, shikimate; SKAP, shikimate 3-phosphate; CHA, chorismate; AGN, arogenate; PPA, prephenate; PPY, phenylpyruvate; ATA, anthranilate.

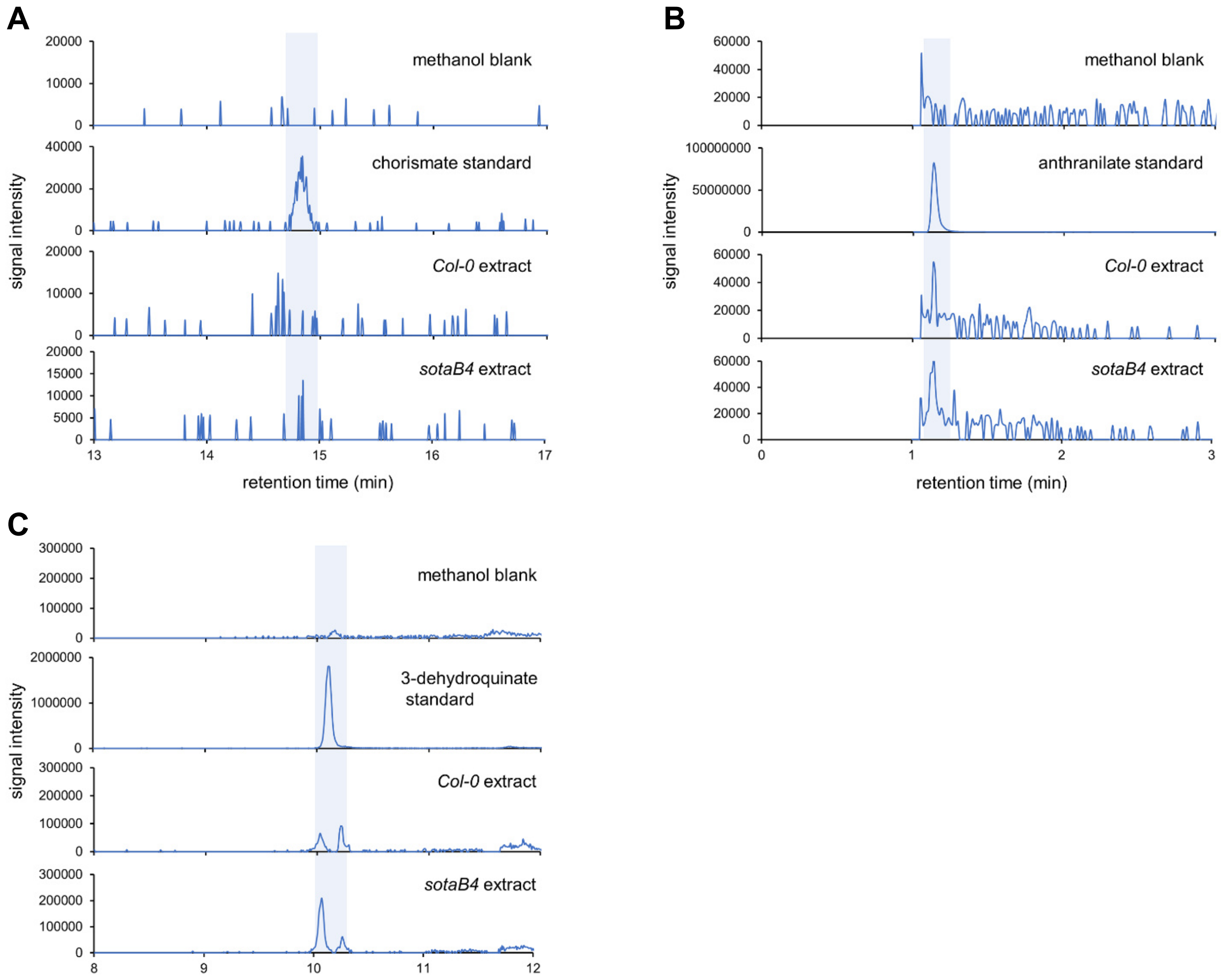

Figure S6. Chorismate, anthranilate and 3-dehydroquinate were poorly detected in Arabidopsis leaf samples. Extracted ion chromatograms corresponding **A)** chorismate, **B)** anthranilate and **C)** 3-dehydroquinate ions in negative mode, as detected in the leaves of Arabidopsis wild type (*Col-0*) and the *sota* mutant line *B4*. Chromatograms correspond to extracts using the non-buffered method, as this method was found to be more efficient to recover these three particular metabolites.

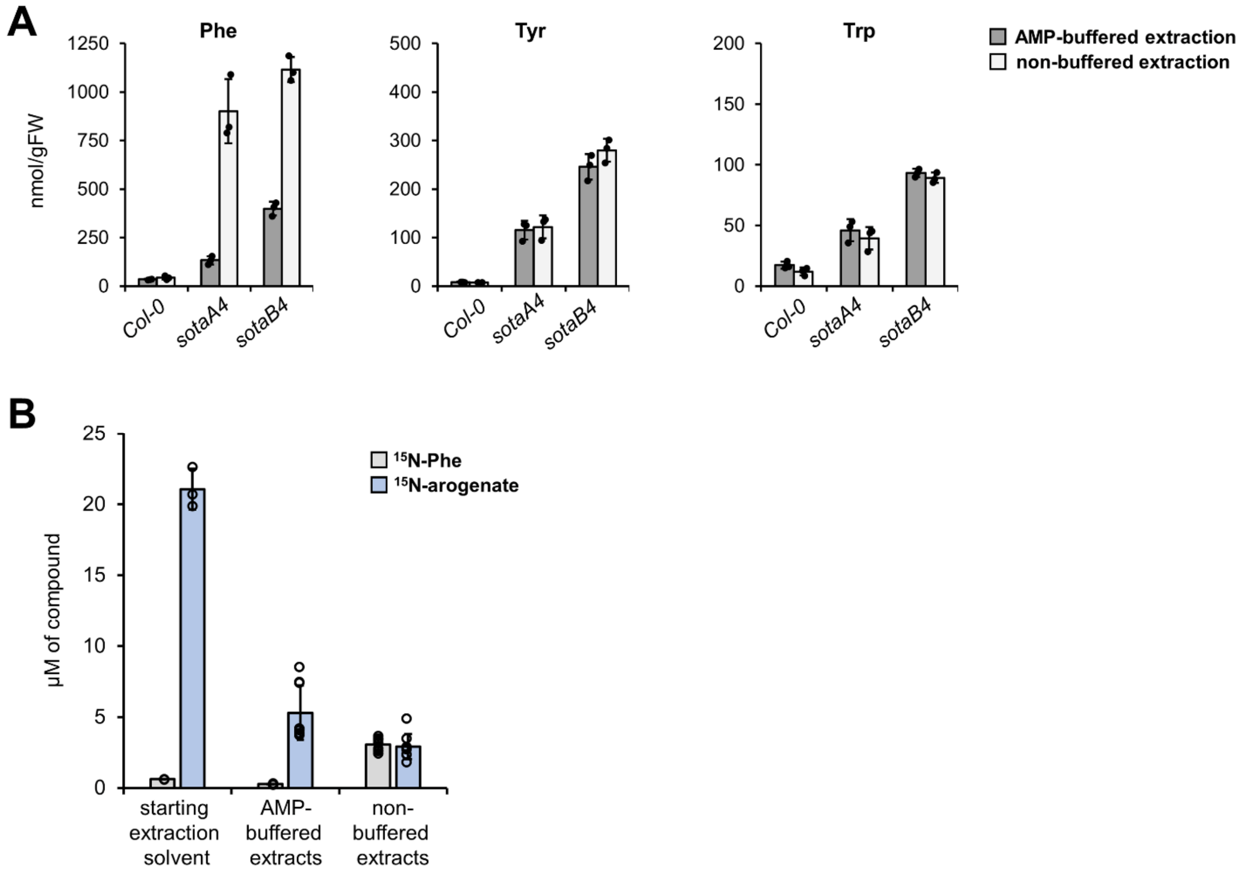

Figure S7. Non-buffered extraction method overestimates Phe content due to arogenate degradation during extraction. A) *In planta* levels of the AAAs in the AMP-buffered extraction compared to the non-buffered extraction, showing higher Phe content in non-buffered extractions compared to AMP-buffered extractions, especially in *sota* lines*.* All three AAAs were corrected by the recovery rate of the norvaline internal standard. Bars represent the average of *n = 3* biological replicates coming from independent plants. Error bars = *SD*. **B)** Levels of ^{15}N-arogenate internal standard and ^{15}N-Phe, presumably produced from ^{15}N-arogenate degradation, in the starting methanol-chloroform mix compared to the final extracts. Bars represent the average of *n = 3* samples for the "starting extraction solvent" (i.e., before any plant material is added) and *n = 9* samples for the "AMP-buffered extracts" and "non-buffered extracts" treatments (final result after completing the extraction procedure, with plant material added). Error bars = *SD*.

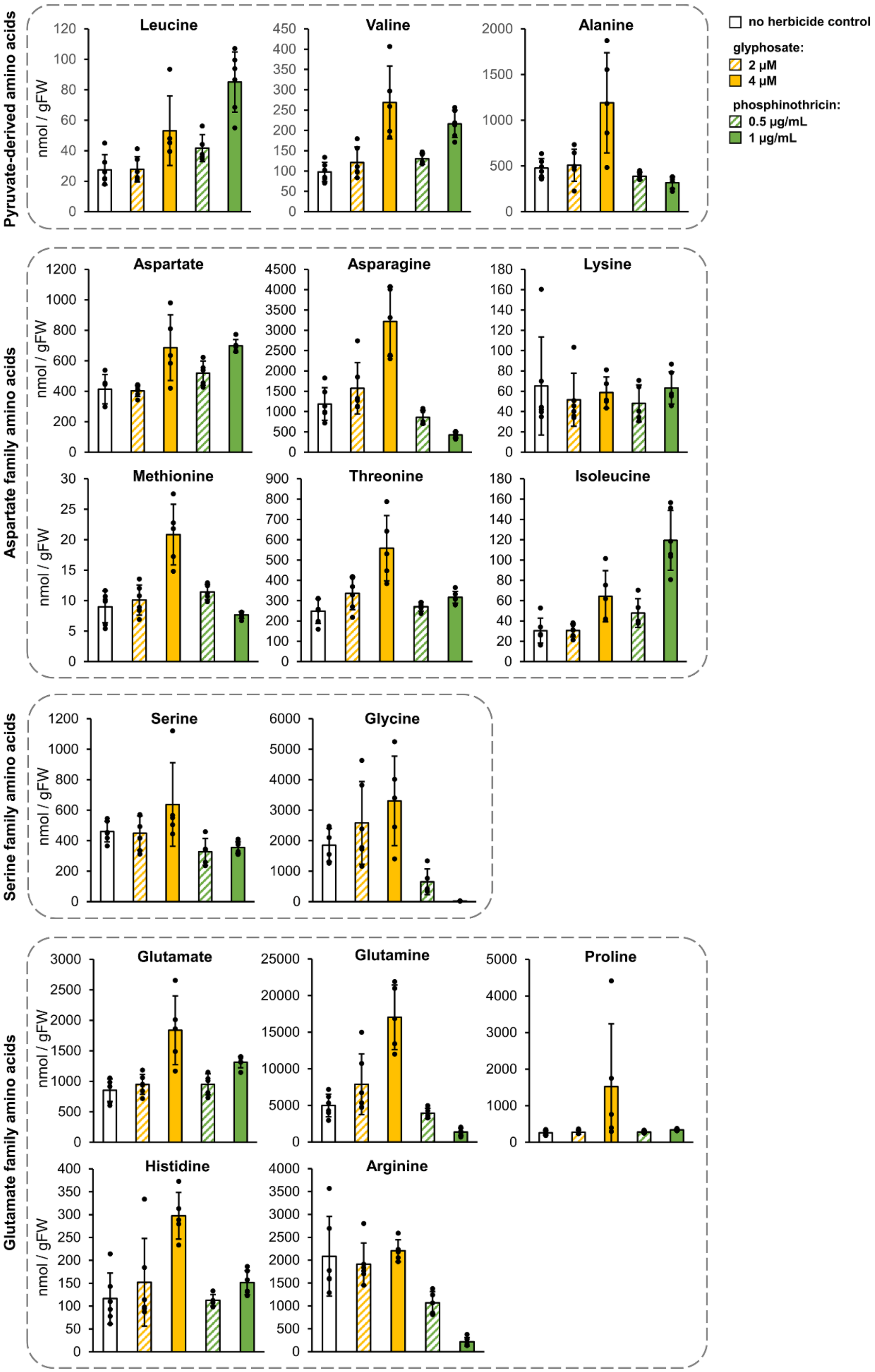

no herbicide control
glyphosate:
2 µM
4 µM
phosphinothricin:
0.5 µg/mL
1 µg/mL
Pyruvate-derived amino acids
Leucine
Valine
Alanine
Aspartate family amino acids
Aspartate
Asparagine
Lysine
Methionine
Threonine
Isoleucine
Serine family amino acids
Serine
Glycine
Glutamate family amino acids
Glutamate
Glutamine
Proline
Histidine
Arginine
nmol / gFW

Figure S8 (previous page). Determination of non-aromatic free amino acid levels in Arabidopsis growth at low doses of glyphosate of phosphinothricin. Bars represent the average of n = 5-6 samples, each corresponding to the whole rosette of an individual plant grown under that treatment. Error bars = *SD*.

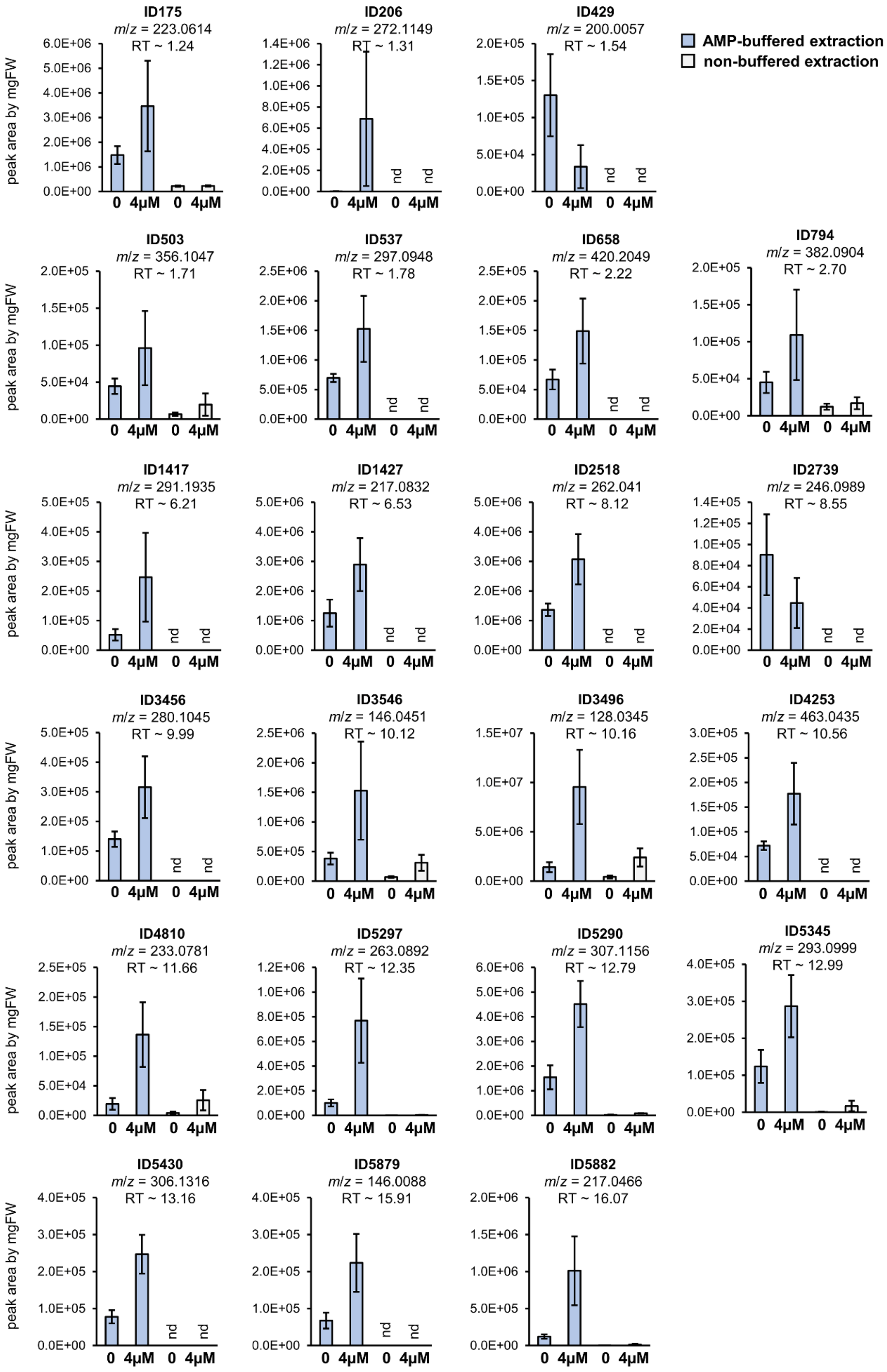
AMP-buffered extraction
non-buffered extraction
peak area by mgFW
ID175
m/z = 223.0614
RT ~ 1.24
ID206
m/z = 272.1149
RT ~ 1.31
ID429
m/z = 200.0057
RT ~ 1.54
ID503
m/z = 356.1047
RT ~ 1.71
ID537
m/z = 297.0948
RT ~ 1.78
ID658
m/z = 420.2049
RT ~ 2.22
ID794
m/z = 382.0904
RT ~ 2.70
ID1417
m/z = 291.1935
RT ~ 6.21
ID1427
m/z = 217.0832
RT ~ 6.53
ID2518
m/z = 262.041
RT ~ 8.12
ID2739
m/z = 246.0989
RT ~ 8.55
ID3456
m/z = 280.1045
RT ~ 9.99
ID3546
m/z = 146.0451
RT ~ 10.12
ID3496
m/z = 128.0345
RT ~ 10.16
ID4253
m/z = 463.0435
RT ~ 10.56
ID4810
m/z = 233.0781
RT ~ 11.66
ID5297
m/z = 263.0892
RT ~ 12.35
ID5290
m/z = 307.1156
RT ~ 12.79
ID5345
m/z = 293.0999
RT ~ 12.99
ID5430
m/z = 306.1316
RT ~ 13.16
ID5879
m/z = 146.0088
RT ~ 15.91
ID5882
m/z = 217.0466
RT ~ 16.07
0 4μM 0 4μM
nd

Figure S9 (previous page). Manual integration of a selection of mass features enriched in the AMP-buffered extraction compared to the non-buffered extraction method. The features, a total of 25, were selected based having the highest MS2 prediction score from those features that changed in response to glyphosate treatment and also were >3-times more abundant in the AMP-buffered extract compared to the non-buffered extract. From the 22 out 25 features analyzed, three could not be found at the expected retention time and therefore were excluded from this figure. Bars represent the average of n = 5-6 samples, each corresponding to the whole rosette of an individual plant grown under that treatment. Error bars = *SD*. n.d. = not detected.
